# Phosphorylation activates the yeast small heat shock protein Hsp26 by weakening domain contacts in the oligomer ensemble

**DOI:** 10.1038/s41467-021-27036-7

**Published:** 2021-11-18

**Authors:** Moritz Mühlhofer, Carsten Peters, Thomas Kriehuber, Marina Kreuzeder, Pamina Kazman, Natalia Rodina, Bernd Reif, Martin Haslbeck, Sevil Weinkauf, Johannes Buchner

**Affiliations:** 1 Center for Protein Assemblies, Department of Chemistry, Technische Universität München, Ernst-Otto-Fischer Str. 8, 85747 Garching, Germany; 2 BNMRZ, Department of Chemistry, Technische Universität München, Ernst-Otto-Fischer Str. 2, 85747 Garching, Germany; 3 Helmholtz-Zentrum München (HMGU), Deutsches Forschungszentrum für Gesundheit und Umwelt, Ingolstädter Landstr. 1, 85764 Neuherberg, Germany; 4 Present Address: Boehringer Ingelheim, Birkendorfer Str. 65, 88397 Biberach an der Riß, Germany; 5 Present Address: Ludwig-Maximilians-Universität München, Biozentrum Großhaderner Str. 2, 82152 Planegg-Martinsried, Germany; 6 Present Address: Roche Diagnostics, Nonnenwald 2, 82377 Penzberg, Germany

**Keywords:** Chaperones, Cryoelectron microscopy

## Abstract

Hsp26 is a small heat shock protein (sHsp) from *S. cerevisiae*. Its chaperone activity is activated by oligomer dissociation at heat shock temperatures. Hsp26 contains 9 phosphorylation sites in different structural elements. Our analysis of phospho-mimetic mutations shows that phosphorylation activates Hsp26 at permissive temperatures. The cryo-EM structure of the Hsp26 40mer revealed contacts between the conserved core domain of Hsp26 and the so-called thermosensor domain in the N-terminal part of the protein, which are targeted by phosphorylation. Furthermore, several phosphorylation sites in the C-terminal extension, which link subunits within the oligomer, are sensitive to the introduction of negative charges. In all cases, the intrinsic inhibition of chaperone activity is relieved and the N-terminal domain becomes accessible for substrate protein binding. The weakening of domain interactions within and between subunits by phosphorylation to activate the chaperone activity in response to proteotoxic stresses independent of heat stress could be a general regulation principle of sHsps.

## Abstract (web summary)

Small heat shock proteins (sHsps) form large spherical assemblies and their regulation is not well understood. Here, the authors provide insights into the mechanism of Hsp26 activation by characterising phospho-mimetic mutants of yeast Hsp26. They present cryo-EM structures of the wild-type Hsp26 40mer and its phospho-mimetic mutants that reveal the location of the thermosensor in the oligomer, and the authors also show that the thermosensor domain is targeted by phosphorylation, which relieves the intrinsic inhibition of chaperone activity.

## Introduction

**Fig. 1:**
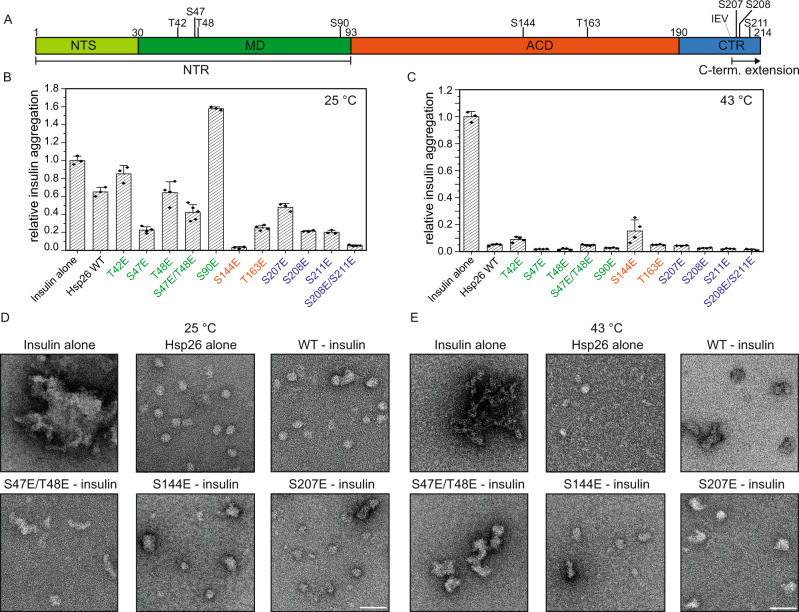
Phospho-mimetic mutants tend to be more active at room temperature. **A** Schematic structure of Hsp26 with highlighted phosphorylation sites. **B** Endpoints of insulin assays performed at 25 °C with 40 µM insulin and 8 µM chaperone concentration are plotted. Data are presented as mean values +/− SD. Single measurement points are indicated in the plot. Aggregation of insulin was induced by the addition of 20 mM DTT. The assay was performed in PBS for 70 min. The light scattering signal (360 nm) was normalized on the saturation value of the model substrate without chaperone. *N* = 3 independent experiments for insulin alone, WT, T42E, S90E, S207E, S208E, and S211E; *N* = 4 for S47E, T48E, S144E, T163E, S208E/S211E; *N* = 5 for S47E/T48E. **C** Endpoints of insulin assays performed at 43 °C with 45 µM insulin and 2 µM chaperone concentration are plotted. Data are presented as mean values +/− SD. Single measurement points are indicated in the plot. The assay was performed in 40 mM HEPES/KOH pH 7.5 for 36 min. The light scattering signal (360 nm) was normalized on the saturation value of the model substrate without chaperone. *N* = 3 independent experiments for insulin alone, S207E, and S208E/S311E; *n* = 4 for all remaining samples. More detailed titrations are shown in Fig. S1. **D** The aggregation assay was performed as described in **B**. For the visualization of chaperone substrate complexes, samples were taken after 20 min and analyzed with TEM at a magnification of 60,000. The scale bar represents 50 nm. **E** The aggregation assay was performed as described in **C**. For the visualization of chaperone substrate complexes, samples were taken after 20 min and analyzed with TEM at a magnification of 60,000. The scale bar represents 50 nm.

Under stress conditions, cells induce the expression of heat shock proteins (Hsps)^1^. Among those are the small (sHsps) which are found in all kingdoms of life and in almost all species^2–5^. sHsps are molecular chaperones with broad substrate spectra^6–9^ suppressing the aggregation of non-native proteins in an ATP-independent manner^9–14^. For refolding of bound substrate proteins, they cooperate with the Hsp70/Hsp40 chaperone system and, in bacteria and yeast, members of the Hsp100 family are additionally involved in this process^9,15–20^. Prominent members of the sHsp family are the α-crystallins, which are abundant in the eye lens^21–23^. Accordingly, the central conserved domain of sHsps is termed α-crystallin domain (ACD). This domain is flanked by an N-terminal region (NTR), which is of variable length and sequence, and a short C-terminal region (CTR)^24,25^. sHsps form large polydisperse oligomers consisting of dimeric building blocks^5,26–28^. The ACD exhibits an immunoglobulin like β-sheet fold and provides the dimerization interface^29–31^. Two different dimerization modes are known. For prokaryotes, archaea, and plants an interaction of the β6 strand of one subunit with the β2 strand of the other subunit was reported and this dimerization mode is referred to as “β6 strand-swapped dimer”^2,17,32,33^. In metazoa, dimerization is mediated by the antiparallel interaction of fused β6 and β7 strands^20,34,35^. In *Saccharomyces cerevisiae*, two sHsps exist, Hsp26 and Hsp42^6,36^. Hsp26 expression is strongly induced upon heat shock and other stresses^37^. Interestingly, the ACD of Hsp26 from *S. cerevisiae* is not sufficient to form stable dimers. It has to be either N-terminally flanked by a part of the NTR, the so-called middle domain (MD) or by the CTR including the IXI motif, which is known to interact with the ACD^2,38^. Both the NTR and CTR mediate the oligomerization of the sHsp dimers into ensembles of larger oligomers^5,32–34,39^. The complexes formed by Hsp26 were reported to range from 24mers to 42mers^10,40–42^. At heat shock temperatures above 40 °C, Hsp26 dissociates into dimers or monomers, which are able to suppress the aggregation of model substrate proteins^10,42^. For this, the NTR, which is subdivided into the MD and a 30 aa long N-terminal sequence (NTS) plays an important role^43,44^. The MD acts as a thermo-sensor, which is essential for the chaperone activity of Hsp26 and mediates conformational changes in the oligomeric complex in response to heat shock temperatures, shifting it from a largely inactive species to an active chaperone^44^. For other sHsps, further activation mechanisms are known. Among them are post-translational modifications such as phosphorylation, changes in pH and hetero-oligomerisation^45–50^. For example, for human Hsp27 and αB-crystallin, three phosphorylation sites in the NTR were shown to decrease the size of the oligomer and to modulate the chaperone activity^50–57^. Also for other chaperone groups, post-translational modifications were shown to be an important regulatory feature^58,59^. For yeast Hsp26, only heat activation has been described so far. Of note, nine phosphorylation sites of Hsp26 were identified in mass spectrometric studies, whereas the structural and functional impact of their phosphorylation on Hsp26 is still unknown^60–69^. They are distributed throughout all structural elements, with some of them forming clusters (Fig. 1A).

In this study, we used phospho-mimetic mutants of Hsp26 to characterize the effect of phosphorylation on Hsp26. The obtained higher-resolution cryo-electron microscopic (cryo-EM) structures of the Hsp26 wild-type (WT) protein and phospho-mimetic mutants allowed us to define the three-dimensional (3D) context of the phosphorylation sites and the mechanism of Hsp26 activation.

## Results

### Phosphorylation-mimetic Hsp26 mutants are more active at lower temperatures

Phospho-proteomic studies had revealed nine phosphorylation sites in Hsp26^60–69^. Strikingly, there are several neighboring sites especially in the MD and the CTR that are phosphorylated (Fig. 1A). For two combinations (S47E/T48E and S208E/S211E), we found evidence in the literature for double phosphorylation. Therefore, the respective double mutants were included in the analysis. It should be noted that also other combinations might occur. Nevertheless, based on the published data, phosphorylation at single sites seems to be preferred. To analyze the potential influence of phosphorylation on the structure and function of Hsp26, we exchanged the respective residues individually against glutamate^70^ thus mimicking the post-translational modification.

To analyze the chaperone activity of the purified proteins in vitro, activity assays with different model substrate proteins were performed (Fig. 1B, C and Supplementary Fig. 1). As the WT protein is largely inactive at lower temperature and becomes active at heat shock temperatures >40 °C^10,71^, we covered a broad temperature range (from 25 to 44 °C) in these chaperone assays. Aggregation of malate dehydrogenase (MDH), which was monitored at 44 °C, was suppressed by the WT and most of the mutants; only a few exhibited lower activities (Supplementary Fig. 1D). This indicates that, at high temperatures, phosphorylation does not negatively affect the activation of Hsp26; however, it also does not activate Hsp26 further. Similar effects were observed analyzing the effects on the aggregation of yeast protein lysate at 42 °C (Supplementary Fig. 1C). In this assay, the S207E mutant was more active than the WT, whereas the activity of the S208E/S211E double mutant was decreased. All other mutants showed activities comparable to the WT.

The aggregation of reduced insulin at 43 °C (Fig. 1C and Supplementary Fig. 1B) was fully suppressed by the Hsp26 WT protein and all variants. However, here, several of the mutants were slightly more effective as chaperones as they suppressed the insulin aggregation already at lower chaperone concentrations (Supplementary Fig. 1B). As this assay is based on the reduction of insulin to trigger aggregation, it can also be performed at lower temperatures. This allowed us to test whether phosphorylation activates Hsp26 already at ambient temperatures. When we performed the insulin aggregation assay at 25 °C, a physiological growth temperature for yeast, the WT protein was almost inactive, as expected^10^. Only a slight reduction of the light scattering signal was observed. Interestingly, phosphorylation-mimetic mutations in the ACD and CTR of Hsp26 suppressed the aggregation of insulin efficiently, indicating that these variants are activated at physiological temperature. Mutants in the MD also suppressed the aggregation of insulin (Fig. 1B and Supplementary Fig. 1A). However, for the S90E mutant, extensive co-aggregation set-in after an initial suppression. For other MD mutants (T42E and S47E/T48E), this co-aggregation effect was also observed but it was less pronounced.

In summary, we conclude that phosphorylation activates the Hsp26 chaperone activity at ambient temperatures; especially the introduction of negative charges in the Hsp26 ACD and CTR induce the activated state. At severe heat stress temperatures, heat activation is the primary modulating factor and the effect of phosphorylation becomes secondary. Depending on the substrate, e.g., for insulin, some of the mutants were still a little bit more efficient in suppressing heat-induced aggregation but this slight activating effect was not observed for all tested substrate proteins and, e.g., in the MDH assay, the mutations rather tended to have a negative effect on the activity at elevated temperatures.

### Phosphorylation-mimetic Hsp26 mutants form stable substrate complexes

To gain further insight into the nature of the chaperoning mechanism of the phosphorylation-mimetic mutants, we analyzed complexes formed with insulin by transmission electron microscopy (TEM). The sHsp/substrate ratios were selected based on the observed activity in the light scattering assays. This allowed us to compare Hsp26–substrate complexes formed upon heat shock and at ambient temperature (Fig. 1D, E). In the control reaction without chaperone, insulin formed aggregates >1 μm at 25 and 43 °C. WT Hsp26 exhibited the expected oligomeric character at 25 °C (Fig. 1D). In the presence of reduced, aggregating insulin, substrate complexes differing in size and shape were formed. Thus, unfolded substrate alone may lead to a slight activation of Hsp26 as also described for other sHsps^45^. In contrast, the Hsp26 variants formed substrate complexes efficiently in agreement with the increased chaperone activity revealed in the substrate aggregation assays. Interestingly, the S47E/T48E mutant formed elongated complexes with reduced insulin at 25 °C. Hence, the secondary increase in the light scattering assays with MD mutants can be explained by a different morphology of the Hsp26–substrate complexes. The substrate complexes formed by the activated S144E mutant as well as the S207E mutant were heterogeneous in size and especially for the S144E mutant big and spherical substrate complexes were observed. Many of the complexes formed by the S207E were comparable to the WT complexes, which is in agreement with the light scattering assay where this mutant was not as strongly activated at 25 °C as the S144E mutant was. In summary, all mutants investigated formed large substrate complexes at 25 °C and the oligomers shrank over time, presumably because Hsp26 subunits were gradually transferred to the growing substrate complexes.

At 43 °C, the Hsp26 WT oligomers dissociated as expected (Fig. 1E). Under these conditions, the shape and size of the substrate complexes formed with either the heat-activated Hsp26 WT or the S207E mutant was similar. Hsp26/substrate complexes were bigger than the Hsp26 oligomer alone and mostly spherical. Interestingly, S144E mutant complexes were smaller. Thus, the amount of insulin that can be integrated into substrate complexes seems to be quite variable, which allows Hsp26 to react in a dynamic range to the presence of unfolded substrates. The substrate complexes with the S47E/T48E mutant were again long-stretched, as observed at 25 °C. This indicates that the negative charges in the MD influence substrate binding and complex formation in a specific manner.

### Phosphorylation-mimetic mutations affect oligomer dissociation

**Fig. 2:**
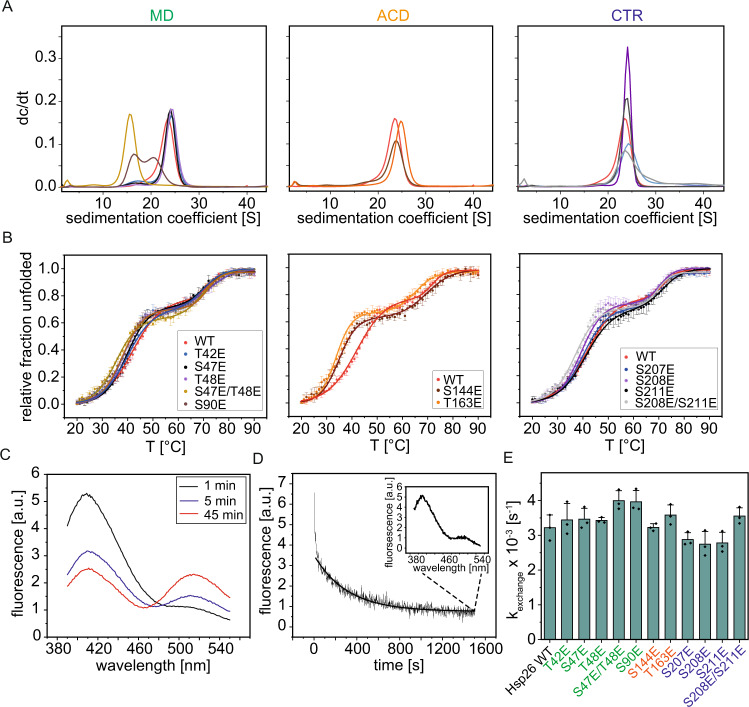
Oligomers of phosphorylation-mimicking mutant dissociate at lower temperatures and tend to be smaller. **A** Determination of the oligomer size by AUC. The proteins were measured at a concentration of 23 µM and 35,000 rpm (98,500 × *g*) and 20 °C in PBS. Sedimentation was followed by UV absorption at 280 nm. Left panel: mutants of the NTR: WT: red, T42E: blue, S47E: black, T48E: purple, S47E/T48E: beige, S90E: brown. Middle panel: mutants of the ACD: S144E: brown, T163E: orange. Right panel: mutants of the CTR: S207E: blue, S208E: purple, S211E: black, S208E/S211E: gray. Runs with different chaperone concentrations are shown in Fig. S2A. HPLC runs are shown in Fig. S2B. **B** Thermal transitions of the proteins were measured in a Chirascan CD spectrometer at 218 nm in PBS. The protein samples (12.5 µM) were heated from 20 to 90 °C. Mean values and the standard deviation are plotted. *N* = 3 independent experiments. The color code is the same as in **A** and is also shown in the graph panels. **C** Formation of Hsp26 FRET pairs. In all, 5 µM AIAS and 5 µM LYI-labeled Hsp26S5C were mixed and incubated at 40 °C. At the indicated time points, spectra were measured at an excitation wavelength of 330 nm. **D** Subunit exchange was measured by the addition of unlabeled Hsp26 to the Hsp26 FRET complex in 39-fold molar excess. The dissociation of the FRET complex was followed at 520 nm (excitation at 330 nm). Subunit exchange was measured at 40 °C for 1500 s. An exemplary dissociation curve obtained for the WT protein is shown. The small inset shows the spectrum after the subunit exchange. **E** Dissociation constants were determined by exponentially fitting the dissociation curves. Mean values and the standard deviation (*N* = 3 independent experiments) are shown. Two-sided Student’s *t* tests were performed to check whether the changes were statistically significant. Only the changes observed for the S47E/T48E mutant were significantly different from the WT (*p* = 0.048).

Since a change in the ensemble of oligomers upon heat activation is a key characteristic of Hsp26^10^, we asked whether the activation of Hsp26 via phosphorylation-mimetic mutations also influences its quaternary structure. Analytical ultracentrifugation (AUC) revealed the presence of large oligomers for WT Hsp26 (23.4S) and the mutant proteins except for two MD mutants (Fig. 2A). The S90E mutant exists in two almost equally populated species, which sediment at 16.4S and 20.5S. Furthermore, the double mutant S47E/T48E forms smaller (15.5S) oligomers than the other variants. The size distribution of the oligomer strongly depends on the concentration of Hsp26 and shifts toward the dimeric species with decreasing protein concentration (Supplementary Fig. 2A) as previously shown for the WT protein^10^. The observed destabilization of the oligomer by phospho-mimetic mutations was further supported by size exclusion chromatography–high-performance liquid chromatography (SEC-HPLC) where many variants except for three CTR mutants (S207E, S208E, and S211E) and the T48E mutant dissociated, due to dilution on the column (Supplementary Fig. 2B).

The interactions governing the oligomeric assembly were also reflected in the thermal stability of the Hsp26 WT. Two transitions can be observed. The higher *T*_M_ represents the unfolding of the ACD while the lower *T*_M_ represents the disassembly of the oligomer^43^. We found that the stabilities of the ACDs were comparable for the WT and the variants, with melting temperatures ranging between 68 and 72 °C (Fig. 2B and Supplementary Table 1). Only the substitution of T163, which is located in the ACD led to a decreased stability (*T*_M_2 = 65.3 °C). However, most of the mutations affected the first transition, the dissociation of the oligomer. The WT protein dissociated at 42.1 °C, which corresponds exactly to the heat shock temperature of *S. cerevisiae*, at which first detrimental effects on cell growth and translation start to set in^72^. Interestingly, except for S47E, S207E, and S211E, all phospho-mimetic mutants exhibited dissociation at temperatures below 40 °C (Supplementary Table 1). As expected from the size analyses before, the S47E/T48E mutant dissociated already at 36.8 °C. In the CTR, only the double mutant S208E/S211E led to a substantial decrease of the dissociation temperature. Notably, the mutations in the ACD led to the strongest decrease of the dissociation temperature (S144E: 35.1 ± 0.1 °C; T163E: 34.6 ± 0.9 °C). This suggests that phosphorylation mainly modulates the stability of the oligomer. As dissociation is linked to activation those results support the assumption that Hsp26 can be activated at temperatures below heat stress. To analyze whether the differences in oligomer stability are also reflected in the dynamics within the oligomer, subunit exchange between Hsp26 complexes was monitored via Förster resonance energy transfer (FRET). To this end, donor- and acceptor-labeled Hsp26 were mixed and incubated at 40 °C (Fig. 2C). After around 30 min, the exchange reaction reached equilibrium. To this preformed FRET complex, unlabeled Hsp26 WT or the respective mutants were added in excess and changes in the acceptor fluorescence were monitored (Fig. 2D). For the WT an exchange constant of 3.2 × 10^−3^ s^−1^ was determined (Fig. 2E and Supplementary Table 1), comparable to previous reports^73^. The exchange constant obtained for the ACD mutants were similar to the WT or slightly increased (S144E: 3.2 × 10^−3^ s^−1^; T163E: 3.6 × 10^−3^ s^−1^). Single substitutions in the CTR seemed to rather stabilize the oligomer and led to a small decrease of the exchange rate. Only for the CTR double mutant (S208E/S211E), an increased exchange rate was determined (3.6 × 10^−3^ s^−1^). For the S47E/T48E mutant (4.0 × 10^−3^ s^−1^; *p* value <0.05), the exchange constant was higher, which correlates to the smaller-sized oligomers. The same was observed for S90E (4.0 × 10^−3^ s^−1^; *p* value >0.05), which also was of smaller size in the AUC experiment. The subunit exchange rates of the other three MD mutants were only slightly increased compared to the WT (3.4–3.5 × 10^−3^ s^−1^).

### The structure of Hsp26 resolved by cryo-EM

**Fig. 3:**
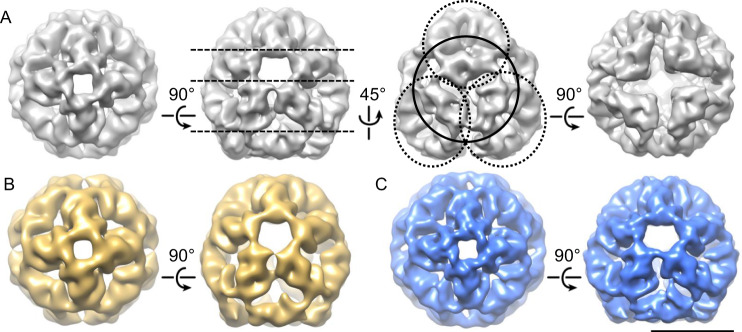
Surface map views of cryo-EM reconstructions. Surface map views of cryo-EM reconstructions thresholded to 83% of the expected volume. **A** Different views (top view, two side views, and a bottom view) of the WT protein (GSFSC resolution 8.2 Å). Top view and bottom view showing fourfold symmetry. The dashed lines in the second view separate the dimer ring stacks. The circle (continuous line) indicates one hexamer while the circles with the dashed line are indicating the three corresponding overlapping hexamers. **B** Two exemplary views of the S47E/T48E mutant 40mer (GSFSC resolution 12 Å). **C** Two exemplary views of the S207E mutant 40mer (GSFSC resolution 7.3 Å). Side view (90° from view A). The length of the scale bar is 10 nm.

To gain insight into the organization of the oligomer, especially the localization of the phosphorylation sites and of the MD, cryo-EM reconstructions of Hsp26 WT and mutants were solved (Supplementary Table S2). Compared to the previous analysis^40^, the new structure revealed that the predominant species of Hsp26 is an oligomer comprising 40 monomers arranged in a raspberry-like shape both for the recombinantly produced proteins (Fig. 3 and Supplementary Fig. 3) and the authentic protein from yeast (Supplementary Fig. 3F). Interestingly, this oligomer structure is shared between the WT and the S207E and S47E/T48E mutants. However, analysis of two-dimensional (2D) class averages revealed that the fraction of particles representing 40mer views was different for the WT and the two mutants. For the WT (48.9%) as well as the S207E mutant (34.2%), the 40mer is the dominant species. While in the S47E/T48E dataset, only 7.3% of the particles could be assigned to 40mer reprojections (Supplementary Table 2). Thus, the mutant assemblies are more heterogeneous concerning the number of subunits incorporated. This is also well reflected in the class averages of the S47E/T48E mutant, which in principle appear like class averages expected for the 40mer. However, some of the density is missing suggesting that subunits are lacking (see marked images in Supplementary Fig. 4E).

**Fig. 4:**
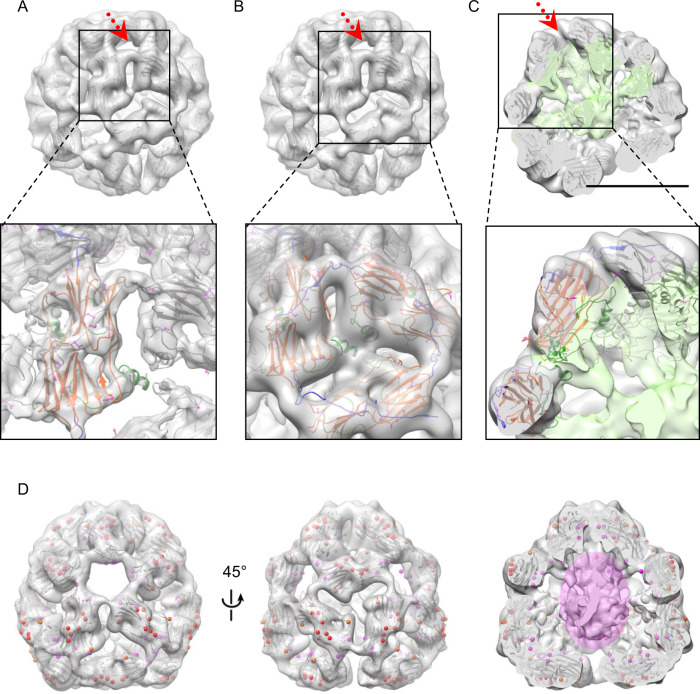
Structure of the atomic model. Each view contains an overview image with a zoomed inset of the highlighted area. The top row overview images contain the WT EM density surface maps with the wheat Hsp16.9 crystal structure (1GME) fitted in. The enlarged area images in the bottom row contain our pseudo-atomic model. **A** Focusing on one dimer in the 40mer environment. The surface model is based on the sharpened partial reconstruction of only the two top rings thresholded to better visualize the loops and the IEV-binding site (GSFSC resolution 6.1 Å). The pseudo-atomic model in the enlarged view at the bottom is color-coded as follows: All potential phosphorylation sites are colored magenta in the ball and stick representation. The ribbon of the central dimer is colored according to the color scheme in Fig. 1A. Thus, the MD is dark green, the ACD orange-red, and the CTR is blue. All other ribbons are colored gray. **B** Focus on one hexamer. Threshold of the surface map rendering was lowered to include the MD and NTS density. Color code as in **A**. The ribbons of all protein chains comprising one hexamer are colored as the central dimer in **A**, while the ribbons of other dimers are colored in gray. **C** Focus on the connectivities of one hexamer and the central core of the structure. The frontal density has been removed to reveal the inner structure. The threshold of the surface map rendering was lowered to include the MD and NTS density as in **B**. The blue C-terminal regions connect dimers at the outside of the oligomer, whereas the MD connects the dimers on the inside. Color-coding was performed as in **A** and the ribbon of the same hexamer is colored as in B. The volume containing middle domain and N-terminus has been highlighted in mint. Scale bar 10 nm. **D** WT 40mer reconstruction with all phosphorylation sites labeled with spheres that are part of the pseudo-atomic model. The sphere is centered around the Cβ atom of the respective residue. For clarity, the C-terminal phosphorylation sites are labeled red and the S144 in orange. The other two sites located toward the inside of the structure are labeled magenta. In the third image, the structure is cut open to show the inside that has been highlighted magenta to roughly indicate the volume encompassing over 60 phosphorylation sites.

The structure of the 40mer has a fourfold symmetric tip, an open bottom (Fig. 3A), and a central core (Fig. 4C). We could resolve the structure of the ACD, the CTR, and parts of the MD. Our pseudo-atomic model of Hsp26 consists of residues 63–214 (71% of the sequence). As can be seen in Fig. 4A, two CTRs bind on top of a dimer forming a ridge over the whole length of the dimer ending in close proximity of each other. This ridge is imbedded on top of the hydrophobic pockets formed by the β4 and β8 strand of the ACD, representing, the IEV-binding site (see enlarged view in Fig. 4A). Most of the density is located in the shell of the structure where the stable, β-sheet structured ACD is located. Depending on the species a sHsp is derived from, this domain can adopt two different forms of dimerization. The non-metazoan type is characterized by an interaction of the β6 strand of one subunit with the β2 strand of the other subunit (β6 strand-swapped dimer), whereas metazoan sHsps dimerize via an antiparallel interaction of fused β6 and β7 strands^2,17,20,32–35^. To better resolve the dimer structure, we concentrated on the top hexamers. A cryoSPARC reconstruction was used to isolate the two top rings of dimers (separated with dashed lines in Fig. 3A). This allowed us to classify the dimers into three differing substructures. The most detailed substructure was used to further refine the reconstruction of this part of the oligomer. This higher resolution structure showed density for the IEV motif bound to the ACD domain and was subsequently used for flexible fitting and modeling (enlarged view in Fig. 4A). Our structural analysis revealed that the ACDs in Hsp26 adopt the non-metazoan association mode, as the loop containing the β6 sheet can be clearly seen in the density maps and fitting of the wheat ACD domain was possible (Fig. 4A). These dimers are the principal building blocks of the 40mer. They associate into hexamers and are connected to other dimers on the outside of the complex through CTR interactions and on the inside through interactions mediated by the MD (Fig. 4). Hence, the NTR of Hsp26, which includes the MD is located in the interior of the structure (Fig. 4C, mint). In contrast to the NTS, our structure reveals that the MD is partially folded and the part adjacent to the ACD could be structurally resolved. Surprisingly, a β-strand of the MD extends the β-sheet of the ACD and then a helix leads further into the core of the structure (Fig. 4B). Thus, the MD is intimately connected to the ACD and three MDs associate with each other. The MDs continue towards the central core (Fig. 4C), which is most likely the position where the NTRs come together.

Within the oligomer, every dimer is part of two of the hypothetical hexamers (see rings in Fig. 4A), because the CTR and MD of each monomer within a dimer interlink in opposite directions. Thus, the wall of the raspberry can be seen as a net in which the ACDs are stitched together by part of the MDs and the CTRs. Within this net, the ACDs have a certain degree of freedom concerning their exact positions. Consequently, dimer conformations and positions are not strictly symmetric and can be different from each other as shown in the 3D classification in Supplementary Fig. 5. In general, for all proteins the size distribution of all class averages matched quite well to that of a perfect 40mer (Supplementary Fig. 5A). Additionally, there are both smaller and larger oligomers present, with the smaller being more abundant in the WT and the S207E mutant. These smaller oligomers could include 24mers. The higher heterogeneity of the S47E/T48E was reflected in its different behavior in the aggregation assays where the MD mutants tended to be activated at lower temperatures but had problems in stably binding the model substrate. The asymmetric 3D classes further prove that Hsp26 can deviate from an “ideal” symmetric structure. This flexibility allows integrating additional dimers such as the one bridging the open bottom (labeled with a red arrow in Supplementary Fig. 5C). In agreement with the asymmetric arrangement and the polarity of the structure, the local resolutions of the individual dimers are different. The dimers located at the tip of the raspberry have a higher resolution than the dimers at the open bottom, which show most variation (Supplementary Fig. 3). As a consequence of the varying flexibility, the density of the dimers varies as well. This can be seen best for the S47E/T48E mutant reconstruction in which the bottom dimers appear smaller than top dimers (side view in Fig. 3B). Higher flexibility ultimately led to lower resolution of parts of the reconstruction (Supplementary Fig. 3). To summarize, the central core is the most flexible part of the structure followed by dimers towards the bottom of the structure and the CTR (arrows in Supplementary Fig. 3D). While the CTRs exhibit some flexibility, their density is always visible even in asymmetric reconstructions indicating that the IEV motif is predominantly bound to the β4/β8 grove of the adjacent dimer. The rather low flexibility of the CTR was also seen by solution-state nuclear magnetic resonance (NMR) of Hsp26. Here only 10 peaks could be observed at 37 °C (Supplementary Fig. 6). Whereas the CTRs of αB-crystallin^74^ and αA-crystallin^47^, which are shown as a comparison, are flexible and could thus be resolved by NMR, it was not possible to observe a high-quality spectrum of the CTR region of Hsp26. Thus, the resolution was not sufficient to obtain a resonance assignment for the CTR of Hsp26.

### Location of the phosphorylation sites within the 40mer

Our structure of the Hsp26 40mer revealed the localization of most of the phosphorylation sites allowing us to rationalize the effects on the organization of the oligomer. The three C-terminal phosphorylation sites (S207, S208, and S211) are near the IEV motif (204–206) of the CTR. Hence, six phosphorylation sites (from the two CTRs) come close together at the ridge that forms the outside connection of neighboring dimers. Their phosphorylation would introduce up to 12 negative charges within this structural element (Fig. 4A, D).

The close association of the MD and the inner side of the ACD brings the phosphorylation site at residue 90 in the MD and the sites at position 144 and 163 into proximity (Fig. 4D), which suggests that phosphorylation at this position might have effects reaching into the MD. The MD furthermore is interlinked with its two counterparts in the middle of the hexamer, oriented towards the inside of the structure (Fig. 4B). Towards the central core, where most of the NTRs come together the phosphorylation sites located at residues T42, S47, and T48 might also come in close contact (Fig. 4D, right panel). Therefore, phosphorylation of those residues could result in extensive rearrangements of the NTR due to electrostatic repulsion.

Taken together, the structure revealed that the phosphorylation sites form patches which are located at strategic positions within the oligomeric structure that upon introduction of negative charges affect the architecture, oligomeric states populated and in consequence the chaperone activity of Hsp26.

### Phospho-mimetic mutations lead to wide-ranged structural changes

**Fig. 5:**
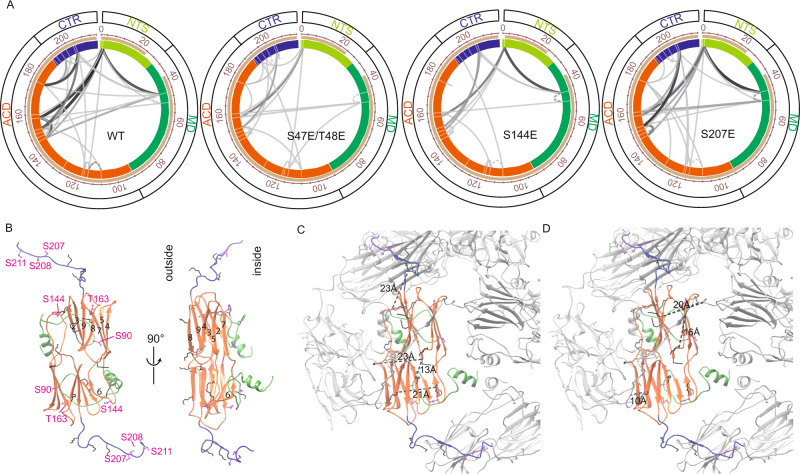
Phosphomimetic mutations lead to an unlocking of the MD. **A** Hsp26 (20 µg) was crosslinked by the addition of 2.7 mM DSG for 1 h at 25 °C. MS measurements were performed on a Thermo Fusion mass spectrometer and the data evaluated in Kojak and visualized with Proxl. The crosslinks are indicated by lines connecting the respective residues. The thickness and color of the lines correspond to the number of peptide spectrum matches (light gray: PSMS = 1–black: PSMS = 5). Dashed lines represent loop-links. White lines in the schematic sequence representation indicate possible crosslink positions. The beige bar represents the sequence coverage. All proteins were measured in triplicates, whereas only one exemplary replicate is shown. **B** Two views of the pseudo-atomic model with the ribbon colored as in Fig. 1A. Potential phosphorylation sites in the stick and ball representation are colored magenta and lysine residues involved in crosslinking as stick model are colored black. **C** Measured intra-dimer crosslinks were mapped on one exemplary dimer and shown as black dashed lines. Distances between the Cα atoms were measured in Chimera and are indicated the pictures. **D** Measured inter-dimer crosslinks are indicated by black dashed lines in the structure segment. Distances between the Cα atoms were measured in Chimera and are indicated in the pictures.

To capture the effect of phosphorylation on the structure in more detail, and to gain more insight into potential interactions between the MD, ACD, and CTR and their dynamics, we performed chemical cross-linking with an amine reactive crosslinker (DSG) at 25 °C and determined the sites connected by mass spectrometry (MS). These experiments revealed that the region from ~aa 44 to 52 in the MD interacts with a patch in the ACD (~144–160) (Fig. 5A) which is part of the long loop containing the β6 strand. Even though the MD could only be modeled to residue 63, the unoccupied electron density had already supported an interaction between the MD and the ACD (Fig. 4C). The crosslinking experiments confirmed this interaction between the MD and the ACD loop, and moreover revealed that this double phosphorylation site seems to be part of the interaction interface. In addition, we found interactions between the ACD and the CTR (~aa 196–204). To test if these interactions are affected by phosphorylation, we also analyzed selected mutants by cross-linking MS. Less cross-linked peptides were found especially for the S47E/T48E and S144E mutants. Here, mainly interactions of the MD with other parts of the protein seem to be weakened suggesting that phosphorylation at those positions has a pronounced impact on the oligomer assembly. In the S207E mutant, the ACD-CTR interaction was hardly affected and the interaction with the MD was only slightly reduced. Plotting the crosslinks into the 3D structure of Hsp26 (Fig. 5B–D) showed that the intra-dimer interactions identified between the CTR and the ACD as well within the ACD fit the structure (Fig. 5C). Furthermore, we were able to map several crosslinks occurring between dimers (Fig. 5D). Due to its dynamics, the resolution of the MD was not sufficient to recapitulate those crosslinks on a pseudo-atomic structural level. We also found interactions of the MD with the CTR in our crosslinking experiments. Based on the cryo-EM structure, those interactions are unlikely to occur in the 40mer and thus rather reflect smaller species interacting with each other. Overall, it appears that interactions tie the MD and the ACD together in the resting state of Hsp26 and that the insertion of a negative charge in the MD or ACD changes the interaction between the structural elements leading to NTR unlocking and activation of Hsp26.

## Discussion

In this work, we set out to understand the effects of phosphorylation on the structure and the activity of Hsp26 from *S. cerevisiae*. Our study revealed that additional negative charges at specific positions in the protein lead to the activation of the Hsp26 chaperone by destabilizing domain interactions in the oligomer.

The phosphorylation-induced decreased stability of the Hsp26 quaternary structure leads to a shift in the ensemble of oligomers towards smaller assemblies. In line with current concepts for the chaperone function of sHsps, these smaller species seem to be more active in binding non-native substrate proteins because they expose binding sites hidden in the larger oligomers. However, it may also be possible to activate the chaperone without disrupting the oligomer especially for phosphorylation of the CTR. Here it seems that the interactions in the oligomer, which intrinsically inhibit the substrate binding is altered in a way that increases local flexibility while keeping subunit contacts largely intact.

Interestingly, mutations in the MD initially delayed the aggregation of the substrate protein insulin and later-on formed co-aggregates with the substrate. The mutations could on the one hand affect conformational transitions in Hsp26 or, on the other hand, directly modulate the interaction with substrates in this region. Based on the crosslinking MS results, it might be both: the negative charge leads to repulsion within the oligomer and liberation of the MD from contacts with the ACD. As the MD is supposed to be an essential substrate interaction site^43^ and the MD mutations decrease its hydrophobicity, this could reduce the binding capacity and/or the potential to keep the unfolded substrate stably bound in solution at non-heat stress conditions. Thus, phosphorylation in the MD might even represent a shift between a stable substrate binding mode (holdase function) and a transient binding mode (foldase function). The MD variants can be additionally heat-activated and behave comparable to the WT at temperatures above 40 °C indicating independent, alternative conformational switches specialized for different stress situations. The mutations in the ACD and CTR already activate Hsp26 at 25 °C and the proteins retain their activity at higher temperatures even though the oligomer size of those Hsp26 variants was hardly affected by the mutations.

Overall, all mutations tested exhibited structural or functional impacts on Hsp26 and therefore none of the sites seems to be a completely silent phosphorylation site, i.e., a site which may be phosphorylated to increase the solubility of the protein but without regulatory function^75,76^.

While phosphorylation allows temperature-independent activation of Hsp26, the principles of the chaperone mechanism seem to be conserved: the large complexes dissociate or change their internal organization. This allows interaction with substrate proteins like insulin to form big assemblies (up to 50 nm)^77^.

**Fig. 6:**
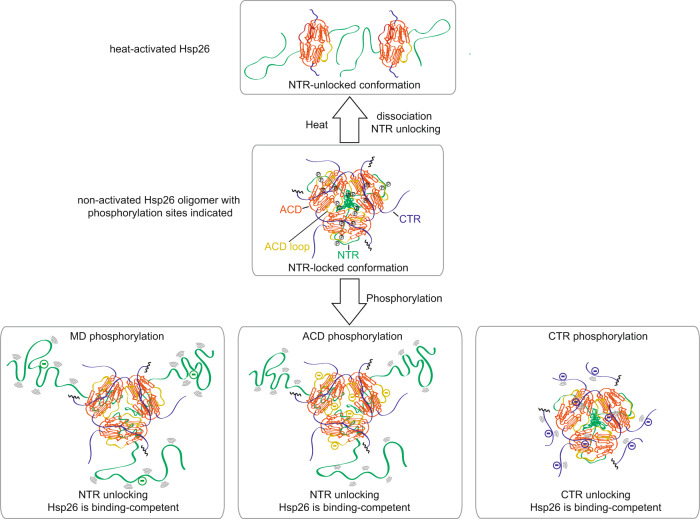
Activation of Hsp26 by phosphorylation-induced unlocking of the MD. In the inactive state, the MD (dark green) interacts with a big loop of the ACD (orange-red). Therefore, it is buried in the oligomer and not able to bind substrate proteins. The MD can be liberated by increasing temperature, which goes along with the dissociation of the oligomer. Alternatively, phosphorylation in specific parts of the MD, ACD, and CTR (blue) can also lead to the unlocking of the NTR. The circle segments represent the dynamics of the NTR or CTR.

Here, we identify the MD-ACD interaction as an important contributor to the regulation of chaperone activity that is targeted by phosphorylation in both domains. Mechanistically, this activation goes along with an exposure of the MD, which is otherwise buried inside the hollow sphere of the inactive 40mer (Fig. 6).

For Hsp26 WT as well as the mutants, a 40mer complex could be resolved. Nevertheless, the mutant protein complexes were more heterogeneous as can be seen in the class averages. Therefore, only a much smaller percentage of particles could be used to reconstruct the 40mer structure. In accordance with the AUC data, this effect was the most pronounced for the S47E/T48E mutant where not even 10% of the particles went into the 40mer.

The 3D structure of the Hsp26 40mer provides insight into the organization of the different structural elements and their interplay. Overall, the oligomer adopts a hollow berry-shaped organization. The ACD dimerizes via β6-swappping, typical for non-metazoan sHsps^20^. The ACD dimers interact with neighboring dimers via the CTRs and NTRs. While the CTRs link the ACDs at the outside of the structure, the NTRs point into the interior of the sphere. Surprisingly, the MD region of the NTR forms a globular structure, which is associated with the ACD extending the ß-sheet by one strand and then leading further into the sphere where the largely unstructured NTS is located. Of note, three MDs come together connecting several subunits in the oligomer. The localization of the MD puts it in the perfect position to mediate regulatory rearrangements in response to external stimuli. Changes in structure will thus efficiently affect the assembly of the entire complex. Based on the 3D structure and the crosslinking data, we suggest an auto-inhibitory locked state in the inactive oligomer where the MD is locked to the loop between the β5 and the β7 strand of the ACD (Fig. 6). The MD, which is the thermosensor and essential for oligomerization^43^, can be either unlocked by temperature or phosphorylation, which renders the entire NTR region accessible to interact with the unfolded substrate. Our results show that MD unlocking can be achieved by phosphorylation in the MD, ACD loop or in the CTR. Thus, while the activating phosphorylation sites in Hsp26 are far apart in the primary sequence they form two patches in the 3D-arrangement of the oligomer; one between the ACD and the MD and the second between two CTRs. For human Hsp27 and αB-crystallin functionally characterized phosphorylation sites are almost exclusively located in the NTR and only one has been analyzed in the ACD^51,56^. Interestingly, however, especially for Hsp27 many more phosphorylation sites have been identified spread throughout the protein^78^ whose function has not been studied yet. Based on our work we suggest a destabilizing effect on the oligomer by 3D-patched phosphorylation also there—as a conserved feature for activation of eukaryotic sHsps with additional regulatory influences depending on the site and the combination of phosphorylation events. As the phosphorylation sites of human sHsps are mostly located in the NTR it is likely that also in this case phosphorylation interferes in the interaction of the NTR and ACD and therefore the mechanism proposed for Hsp26 might be conferrable to mammalian sHsps at least to some extent. We suggest that phosphorylation of Hsp26 or sHsps in general is an important tool in response to other proteotoxic stress conditions such as oxidative stress, ethanol stress, exposure to toxic other substances or defects in protein translation. This has to be studied further potentially also with other sHsps as Hsp26 is not essential and its deletion does not lead to a clear phenotype^36,79^.

## Methods

### Design of phosphorylation mutants

Due to initial cloning of HSP26 into pQE60 with *Nco*I and *Hind*III, an additional glycine after the start methionine was inserted. Nevertheless, for numbering of the amino acids in the scheme, the WT sequence without glycine was used. Point-mutations were generated using Q5 site-directed mutagenesis (NEB). The mutations were encoded in the respective primers (primer sequences are listed in Supplementary Table 3) and the whole vector (pQE60::HSP26) was amplified via PCR. In all, 50–100 µg DNA were treated with 1 µl DpnI, 1 µl T4 PNK, and 1 µl T4 DNA ligase for 60 min at room temperature (RT). The ligation product was transformed into chemocompetent *Escherichia coli* XL1 Blue cells and the sequence was verified by Sanger sequencing.

### Expression and purification of Hsp26 and phosphorylation-mimetic mutants

*E. coli* HB101 was transformed with pQE60::HSP26 plasmids. Two liters of LB_Amp_ were inoculated with the respective *E. coli* overnight culture and the cells grown at 37 °C until an OD_600_ of 0.8 was reached. Protein expression was induced by the addition of 1 mM IPTG and took place overnight at 37 °C. On the next day, the cells were harvested in a Beckman Avanti J-26 XP centrifuge (JA-10 rotor) for 20 min and 5000 × *g* at 4 °C. The pelleted cells were washed one time in 30 ml ice-cold buffer A (20 mM HEPES, 50 mM KCl, 5 mM EDTA, pH 7.5). After cell disruption with a French press (Constant systems, Basic Z), the clarified lysate was loaded onto an equilibrated anion exchange column (Q-Sepharose). The column was washed with 5 column volumes (CV) buffer A and eluted by anion displacement (300 ml linear gradient until 20 mM HEPES, 525 mM KCl, 5 mM EDTA, pH 7.5) at a flow rate of 2.8 ml/min. Sodium dodecyl sulfate–polyacrylamide gel electrophoresis-selected fractions were pooled and concentrated to 10 ml in a stirred cell (Amicon) with a 10 kDa cut-off filter. The concentrated protein solution was loaded onto Superdex200 26/60 and run at 1 ml/min with buffer A. Selected fractions were pooled and loaded on a Resource Q (6 ml, GE) equilibrated in buffer A. The column was washed with 100 ml buffer A and then eluted by increasing concentration of buffer B (100 ml gradient until 50% buffer B). The proteins were purified at 4 °C. Fractions of sufficient purity were combined, dialyzed against phosphate-buffered saline (PBS), concentrated, flash frozen in liquid nitrogen, and stored at −20 °C.

### Far ultraviolet (UV) spectroscopy—thermal transitions

CD spectra were recorded in 0.5 mm cuvettes (Hellma) on a Chirascan™–Circular Dichroism Spectrometer (Applied PhotoPhysics) at a concentration of 12.5 µM in PBS. Far UV spectra were measured from 280 to 200 nm. Thermal transitions were measured from 20 to 90 °C with a heating rate of 1 K/min. The transition at 218 nm (mean of three replicates) is shown in the plots. The thermal transition temperatures were determined using the Boltzmann Fit in OriginLab (Eq. 1). In Table S1, the mean values of the three independent replicates are shown. For the fits in Fig. 2, a double Boltzmann Fit (Eq. 2) was applied.

1 $$y={A}_{2}+\frac{{A}_{1}-{A}_{2}\,}{1+{e}^{(x-{x}_{0})/{{{{{{\rm{d}}}}}}x}}}$$

2 $$y={y}_{0}+A * ({{{{{\rm{frac}}}}}}/(1+{{\exp }}((x-{x}_{01})/{k}_{1}))+(1-{{{{{\rm{frac}}}}}})/(1+{{\exp }}((x-{x}_{02})/{k}_{2})))$$

### Förster resonance energy transfer

For labeling of Hsp26 S5C, the protein (in PBS) was reduced by the addition of 0.5 mM TCEP for 1.5 h at RT. Subsequently, the lucifer yellow iodoacetamide (LYI; Invitrogen) or 4-acetamido-4′-((iodoacetyl)amino)-stilbene-2,2′-disulfonic acid (AIAS; Life Technologies) dye were added in 10-fold molar excess and the reaction was incubated at RT overnight and for 3 more hours at 37 °C in the dark^80^. The free labels were removed with PD10 columns. For FRET measurements, 5 µM of each labeled protein were mixed in a 200 µl cuvette. The experiment was performed in PBS at 40 °C. The fluorescence donor was excited at 330 nm and the spectra were measured from 390 to 550 nm at the indicated time points.

### Subunit exchange measurements

The Hsp26 FRET complex with 5 µM LYI-labeled and 5 µM AIAS-labeled Hsp26 S6C was pre-formed for 1 h at 40 °C. The unlabeled protein solutions were also adjusted to 10 µM and incubated for 1 h at 40 °C. The exchange reaction was started by the addition of 5 µl of the FRET complex to 195 µl of unlabeled protein. The decrease of the acceptor fluorescence at 520 nm (excitation: 330 nm) was followed in a Fluoromax4 spectrofluorometer (Horiba Jobin Yvon) at 40 °C for 1500 s. The decay curve was fitted with an exponential decay fit (Eq. 3). The exchange constant was calculated with Eq. 4. The mean values and the standard deviation from three independent replicates were calculated.

3 $$y={A}_{1}\times {e}^{-\frac{x}{{t}_{1}}}+{y}_{0}$$

4 $${k}_{{{{{{\rm{exchange}}}}}}}=1/{t}_{1}$$

### Insulin aggregation assay

25 °C: PBS, Hsp26 (0, 2, 4, 8 µM) and 40 µM Insulin (Sigma) were mixed in a cuvette (volume: 196 µl). The solution was incubated for 5 min at 25 °C in a photometer (Cary 50; Jasco) equipped with a temperature-adjustable cell holder. To reduce insulin and to initiate aggregation, 4 µl 1 M dithiothreitol (DTT) were added. After the addition of DTT, the solutions were mixed again and light scattering was measured at 360 nm for 70 min (CaryWinUV Bio pack software version 3.0). WT/0 µM, S47E/0 µM/4 µM/8 µM, T48E/0 µM//8 µM, S47E/T48E/0 µM, S90E/4 µM, T163E/4 µM/8 µM, S207E/0 µM/4 µM, S208E/0 µM, S211E/0 µM, and S208E/S211E/0 µM/4 µM were measured in four independent replicates. S47E/T48E/4 µM/8 µM were measured in five independent replicates. The rest was measured in independent triplicates.

43 °C: 40 mM HEPES/KOH (pH 7.5), Hsp26 (0, 0.5, 1, 2 µM) and 45 µM Insulin (Sigma) were mixed in a cuvette (volume: 197 µl)^10^. The solution was incubated for 10 min at 43 °C in a photometer (Cary 50; Jasco) equipped with a temperature-adjustable cell holder. To reduce insulin and initiate aggregation, 3 µl 1 M DTT were added. After the addition of DTT, the solutions were mixed again and light scattering was measured at 360 nm for 36 min (CaryWinUV Bio pack software version 3.0). WT/0 µM/2 µM, T42E/2 µM, S47E/2 µM, T48E all concentrations, S47E/T48E/1 µM/2 µM, S90E/2 µM, S144E/0.5 µM/1 µM/2 µM, T163E/0 µM/2 µM, S207E/1 µM, S208E all concentrations, S211E/0.5 µM/1 µM/2 µM, and S208E/S211E/0 µM/0.5 µM/1 µM were measured in four independent replicates. The rest was measured in independent triplicates.

### MDH aggregation assay

Hsp26 (0, 0.5, 1, 2 µM) was diluted in PBS in a cuvette and incubated for 10 min at 44 °C. The reaction was started by the addition of 2 µM MDH (pig heart; Roche) and followed for 70 min^8^ with the CaryWinUV Bio pack software version 3.0. Activity comparisons were performed with 1.3 µM chaperone. S47E/0 µM, S47E/T48E/1 µM, S144E/0 µM /0.5 µM/1 µM, S207E/0 µM, S211E/0.5 µM/2 µM, and S208E all concentrations were measured in independent triplicates. The rest was measured in four independent replicates.

### Lysate aggregation assay

Cells (BY4741 *hsp26∆*) were grown in 400 ml YPD from OD_595_ 0.2 to OD_595_ 0.9 at 25 °C. Subsequently, the cells were harvested at 7000 × *g* for 10 min and 4 °C (Beckman Coulter) and the pellets washed one time with 40 ml PBS (4163 × *g* for 10 min and 4 °C; Hettich). Then the cells were resuspended in 5 ml PBS supplemented with 1 mM DTT, 1:100 protease inhibitor MixFY (Serva), and 1:100 Phosphatase Mix 2 and 3 (Sigma). For cell lysis with glass beads in a mixer mill (Retsch; 4 × 90 s at 30 Hz), the suspension was aliquoted into 2 ml tubes. The cell debris was pelleted for 10 min at 16,000 × *g* and 4 °C and the supernatants were combined. Cellular ATP was degraded by the addition of hexokinase (Roche) to a final concentration of 30 U/ml together with 2 mM MgCl_2_. The reaction was incubated for 20 min at 20 °C. Subsequently, the protein concentration was determined via BCA assay; 300 µl aliquots (5.3 mg/ml) were frozen in liquid nitrogen and stored at −80 °C. After thawing, the protein solution was centrifuged for 10 min at 16,000 × *g* and 4 °C to remove aggregates. The assay volume was 50 µl with a lysate protein concentration set to 0.8 mg/ml and 1 mM DTT. The sHsps were activated for 60 min at 42 °C, titrated to the lysate (0, 0.5, 1, 2, 4, 6, 8, 12, 16 µM) and the mixtures were incubated for 90 min at 42 °C. Then the samples were centrifuged for 10 min at 8600 × *g* and 4 °C and the supernatant was taken off. The pellets were washed two times with 100 µl ice-cold PBS. Finally, they were dissolved in 40 µl 1× Laemmli buffer and boiled for 5 min at 95 °C. In all, 22 µl were loaded on 4–20% gradient gels (Serva) and run at 20 mA per gel. The gels were stained with Coomassie Quick Stain (Serva) for at least 2 h and destained with water while shaking. Densitometric analysis was performed with the ImageQuant TL software (GE). For the WT, 5 independent replicates were measured, for the S208E mutant 4 replicates, and for the other mutants 3 replicates were measured (T42E, 8 µM was also measured 4 times).

### Solution-state NMR

Solution-state NMR experiments were performed with Bruker NMR spectrometers operating at magnetic field strengths of 14.1 T. The spectrometer was equipped with cryogenically cooled probe. Solution-state ^1^H–^15^N HSQC experiments with [^13^C, ^15^N]Hsp26 were performed at 37 °C in PBS (pH = 7.4) containing 10% D_2_O. The concentration was in the range of 0.04–0.08 mM (monomer concentration).

### Negative stain EM of aggregation assays

Insulin aggregation assays were performed as described above and 5 µl samples were taken before start and after 20 min. Copper grids with continuous carbon film were glow discharged for 30 s and 5 µl of the sample of the chaperone assay were adsorbed for 20 s (after 1:10 dilution for the higher concentrated 25 °C sample). Subsequently, the grids were washed with 20 µl HEPES buffer (40 mM HEPES, 50 mM KCl, pH 7.4) and stained with 5 µl 2% uranylacetate solution for 30 s. Micrographs were taken with a Ruby camera installed in a JEOL 1400 plus microscope operated at 120 kV at a nominal magnification of 60 k, which resulted in a pixel size of 0.275 nm/px. Scale bars were added and images were cut and rescaled with Gimp (version 2.10.22).

### Analytical SEC-HPLC

Analytical size exclusion columns (Superdex200 increase 10/300GL; GE) were operated with a Shimadzu HPLC system. The proteins were detected by UV absorption with an SPD-20A detector (Shimadzu) and a fluorescence detection system RF-10A XL (Shimadzu). Furthermore, the HPLC system was equipped with a SIL-20AC auto sampler (Shimadzu) and a DGU-20A degasser unit (Shimadzu). The retention times of the investigated proteins were compared with the retention times of standard proteins from the BioRad gel filtration standard (BioRad), which contains Thyroglobulin (670 kDa), γ-Globulin (158 kDa), Ovalbumin (44 kDa), Myoglobin (17 kDa), and Vitamin B12 (1.35 kDa). The HPLC runs were performed in PBS at a flow rate of 0.5 ml/min and at RT. Twenty microliters of a 20 µM protein solution were loaded. One exemplary run of three independent runs is shown in the figure.

### Analytical ultracentrifugation

AUC measurement were carried out using a ProteomLab XL-I (Beckman) equipped with absorbance optics. The protein concentrations for the measurements were 23, 7, and 2.3 µM in PBS, respectively. The samples (350 µl) were loaded into assembled cells with quartz windows with 12-mm-path-length charcoal-filled epon double-sector centerpieces. The measurement took place at 35,000 rpm in an eight-hole Beckman-Coulter AN50-ti rotor (equates 98,500 × *g*) at 20 °C. Sedimentation was continuously scanned with a radial resolution of 30 µm and monitored at 280 nm. Data analysis was carried out with SEDFIT using the continuous c(S) distribution mode^81,82^.

### Cross-linking MS—sample preparation

The applied crosslinking protocol is based on a recent published protocol with several modifications^83^. Twenty micrograms of Hsp26 (in 40 µl PBS) were incubated for 1 h at 25 °C. Then 4 µl of freshly prepared 30 mM disuccinimidyl glutarate (ThermoFisher Scientific; in dimethyl sulfoxide) were added and the cross-linking reaction took place for 1 h at 25 °C. The reaction was stopped by the addition of 6 µl 1 M Tris/HCl (pH 7.4). 29.5 µl 6 M Urea, 50 mM Tris/HCl (pH 7.4), and 2.7 µl 100 mM DTT were added and the samples were incubated for 30 min at 56 °C to denature the crosslinked proteins. After cooling the samples back to RT, 6.3 µl 100 mM iodoacetamide were added and the samples were incubated for 20 min at RT in the dark. Subsequently, the proteins were digested by the addition of 1 µl sequencing grade Trypsin (500 ng; Promega) overnight at 37 °C. The digest was stopped by the addition of 1 µl formic acid (FA). The samples were desalted with double C18 layer stage tips^84^. To this end, the tips were equilibrated with 70 µl methanol and washed three times with 70 µl 0.5% FA by applying mild centrifugation steps (960 × *g*). The peptides were applied onto the stage tips, washed three times with 70 µl 0.5% FA, and eluted with two times 30 µl 80% ACN, 0.5% FA (960 × *g*). The samples were dried in a speed vacuum concentrator (Eppendorf). For the MS/MS measurement, the peptides were dissolved in 25 µl 0.5% (v/v) FA and incubated for 15 min in an ultrasonic bath at RT. The peptide solutions were filtered with centrifugal filters (0.22 µM; Merck; 1 min at 7000 × *g*) and transferred into Chromacol vials (ThermoFisher Scientific).

### Cross-linking—MS/MS measurement

MS/MS measurements were performed on an Orbitrap Fusion instrument coupled to an Ultimate3000 Nano-HPLC via an electrospray easy source (ThermoFisher Scientific). After loading the peptides on a 2 cm PepMap RSLC C18 trap column (particles 3 µm, 100 Å, inner diameter 75 µm, ThermoFisher Scientific) with 0.1% TFA, they were separated on a 50 cm PepMap RSLC C18 column (particles 2 µm, 100 Å, inner diameter 75 µm, ThermoFisher Scientific) constantly held at 40 °C. The peptides were eluted with a gradient from 5 to 35% ACN, 0.1% FA during 35 min at a flow rate of 0.4 µl/min (7 min 5% ACN, 30 min to 28% ACN, 5 min to 35% ACN, 0.1 min to 90% ACN, 10 min wash at 90% ACN, 10 min equilibration at 5% ACN).

Survey scans (*m*/*z* 300–1500) with a resolution of 120,000 were acquired. The automatic gain control (AGC) target value was set to 2.0*e5 with a maximum injection time of 80 ms. For HCD fragmentation, the most intense ions of charge states 2–12 were selected. The collision energy was set to 30%. In the ion trap, the maximum injection time was set to 100 ms and the AGC target value was reduced to 5.0*e4. Inject ions for all available parallelizable time was allowed. Dynamic exclusion of sequenced peptides was set to 60 s. Internally generated fluoroanthene ions were used for real-time mass calibration. Data were acquired using the Xcalibur software version 3.0sp2 (ThermoFisher Scientific). The experiments were performed in three independent replicates. One exemplary replicate is shown in the figure.

### Cross-linking MS data evaluation

Converted raw files (*.mzXML*) were processed with Kojak (version 1.6.1)^85^. The crosslinks were visualized with ProXL^86^. In the PSM filter section the score (Kojak) was set to 2.0 and the min. PSM value was set to 1.

### Cryo-electron microscopy

Four microliters of protein solution (Hsp26 WT recombinant or purified, S47E/T48 and S207E mutants) were applied to glow-discharged Quantifoil R2/1 or R1.2/1.3 holey carbon grids and plunge-frozen in an ethane/propane mixture (26% ethane) using a Thermo Fisher Vitrobot Mark IV (force 10). Protein concentration and blotting time were adjusted to optimize ice thickness and distribution for automated imaging. The protein concentration ranged between 2.0 and 3.3 mg/ml and the blotting time ranged between 2 and 8 s. The grids were transferred into a Titan Krios microscope (Thermo Fisher) equipped with a GATAN K2 summit camera operating in counting mode. The microscope was operating with 300 kV in the energy-filtered TEM mode. Automated image acquisition was performed with SerialEM^87^, collecting 3 micrographs per hole for the R2/1 grids and one image per hole for the R1.2/1.3 grids. The nominal magnification was set to 105 k and the nominal defocus was set between 0.6 and 2.8 µm. The electron dose ranged between 1.6 e/Å^2^ per second and 5.7 e/Å^2^. Five frames per second were collected and the total exposure time was set to 10 s when the dose was below 3 e/Å^2^ s and to 8 s in all other cases. See Table S2 for dose settings and the numbers of micrographs and particles for the different samples. The final pixel size was 1.33 Å/pix.

### Image processing

Frames were aligned and dose weighted with MOTIONCOR2 (number of patches 9 × 9). The resulting images were analyzed by Gctf (v 1.06). Micrographs were discarded if the max. resolution was worse than 4.2 Å or if defocus was outside the range between 0.5 and 2.8 µm or if astigmatism was worse than 800 or if the figure of merit was better than 9999 (which indicated carbon instead of ice). WT particles were picked with gautomatch (version 0.53, developed by Dr. Kai Zhang, MRC Laboratory of Molecular Biology, Cambridge, UK) or with crYOLO (version 1.5.6)^88^. The default crYOLO model was fine-tuned with 16 manually picked micrographs. Particles were extracted with Relion (version 3.2.0)^89^ with a boxsize of 288 px. Falsely picked particles were removed by 2D classification in Relion. The remaining particle numbers are listed in Supplementary Table 2.

### 2D analysis

These particles were then imported into IMAGIC5^90^, which was used for all 2D analysis steps including the Multivariate Statistical Analysis (MSA). Class averages generated by MSA are more varied and detailed overall than 2D averages produced by Relion or cryoSPARC. Particles were binned by factor 2, filtered normalized and centered. Centered particles were used to generate free reference class averages using MSA with 500 images per class on average (class average number varied between 321 and 1554 depending on dataset size). The ferret diameter of all class averages was determined manually with ImageJ and that diameter was assigned to all class members. The assigned diameters were rounded to 0.5 nm and plotted as a size distribution histogram. Four hundred and eighteen equally distributed reprojections were used in the same way to generate the size distribution of a pure 40mer dataset. To determine which of the class averages represent 40mer views, we used cross correlation between the class averages and the reference reprojections. To this end, class averages were masked and aligned to the reference with the highest cross correlation efficient. We then inspected the class averages alongside the corresponding reference to decide whether this indeed represented a 40mer view or not.

### 3D reconstruction

Particles were imported into cryoSPARC (version 3.2.0)^91^ and all subsequent processing steps utilized cryoSPARC. Ab initio reconstruction was used to test which symmetry works best. Reconstructions with discernible dimer structures were achieved only with C1, C2, and C4 symmetry. The best class from ab initio reconstructions was then further refined by heterogeneous refinement and non-uniform homogenous refinement or default homogenous refinement to generate the 40mer structures presented in this study. These structures were still heterogeneous as evident from heterogeneous refinement (3D classification) and 3D variability analysis. To improve the dimer structure, symmetry expand was used on the highest resolution map (S207E), followed by local refinement and particle subtraction to focus on reconstructing of only a part comprising eight dimers, which was used for the flexible fitting. 3D structures were visualized with Chimera (version 1.15)^92^.

### Structure modeling

I-Tasser^93^ was employed to generate a homology model of the Hsp26 structure based on the wheat 1GME structure. Only the part of the sequence that we were confident to fit in flexibly was used. Finally, it contained residues 63–214. MODELLER^94^ integration in Chimera was used to complete the loops of the CTR connecting different dimers. NAMD (version 2.12)^95^ was used for the MDFF simulations, which was controlled and visualized inside VMD^96^. Simulations were set to simulate the protein structure in vacuum at a temperature of 300 K and a grid force of 0.3. This was done iteratively with simulation times of 10,000 and 2000 fs of energy minimization. For the last iteration, the grid force was ignored. The quality of the model was assessed in Phenix (version 1.19.2)^97^.

### Statistics and reproducibility

Mean values and the standard deviation were calculated with Origin 2018b. For statistical analysis, two-sample *t* tests were performed with Origin 2018b. For the visualization of the crosslinking MS data, all identified peptides were taken into account. For negative stain and cryo-EM, multiple grids were prepared with varying concentrations and plunging conditions. Each experiment was repeated at least two times except for the WT yeast control. Negative stain images included in the paper were selected from a set of 20–30 images.

### Reporting summary

Further information on research design is available in the Nature Research Reporting Summary linked to this article.

## Supplementary information

Supplementary Information

Reporting summary

## Data Availability

Cryo-EM density maps have been deposited in the EMBD under the accession codes EMD-12773 (WT), EMD-13748 (WT yeast), EMD-12772 (S47E/T48E), EMD-12771 (S207E), and EMD-12766 (map of the top two rings used for flexible fitting). The coordinates for the atomic model have been deposited in the PDB database under accession code 7OA6. The mass spectrometry proteomics data have been deposited to the ProteomeXchange Consortium via the PRIDE^98^ partner repository with the dataset identifier PXD025314. Source data are provided with this paper.

## Source data

Source Data
