# Supplementary material for: Phosphorylation activates the yeast small heat shock protein Hsp26 by weakening domain contacts in the oligomer ensemble
Source: Nat Commun. 2021 Nov 18;12:6697. doi: 10.1038/s41467-021-27036-7 (PMC8602628; doi:10.1038/s41467-021-27036-7)
Supplement: Supplementary file 1 — Supplementary Information [file 41467_2021_27036_MOESM1_ESM.pdf]

Supplementary Information

Phosphorylation activates the yeast small heat shock protein Hsp26 by weakening domain contacts in the oligomer ensemble

Moritz Mühlhofer[1#], Carsten Peters[1#], Thomas Kriehuber[1,4], Marina Kreuzeder[1,5], Pamina Kazman[1,6], Natalia Rodina[2,3], Bernd Reif[2,3], Martin Haslbeck[1], Sevil Weinkauf[1], Johannes Buchner[1*]

[1] Center for Protein Assemblies, Department of Chemistry, Technische Universität München, Ernst-Otto-Fischer Str. 8, 85747 Garching, Germany

[2] BNMRZ, Department of Chemistry, Technische Universität München, Ernst-Otto-Fischer Str. 2, 85747 Garching, Germany

[3] Helmholtz-Zentrum München (HMGU), Deutsches Forschungszentrum für Gesundheit und Umwelt, Ingolstädter Landstr. 1, 85764 Neuherberg, Germany

[4] current address – Boehringer Ingelheim, Birkendorfer Str. 65, 88397 Biberach an der Riß, Germany

[5] current address – Ludwig-Maximilians-Universität München, *Biozentrum* Großhaderner Str. 2 82152 Planegg-Martinsried, Germany

[6] current address – Roche Diagnostics, Nonnenwald 2, 82377 Penzberg, Germany

# contributed equally

* corresponding author: johannes.buchner@tum.de

Supplementary Figure 1

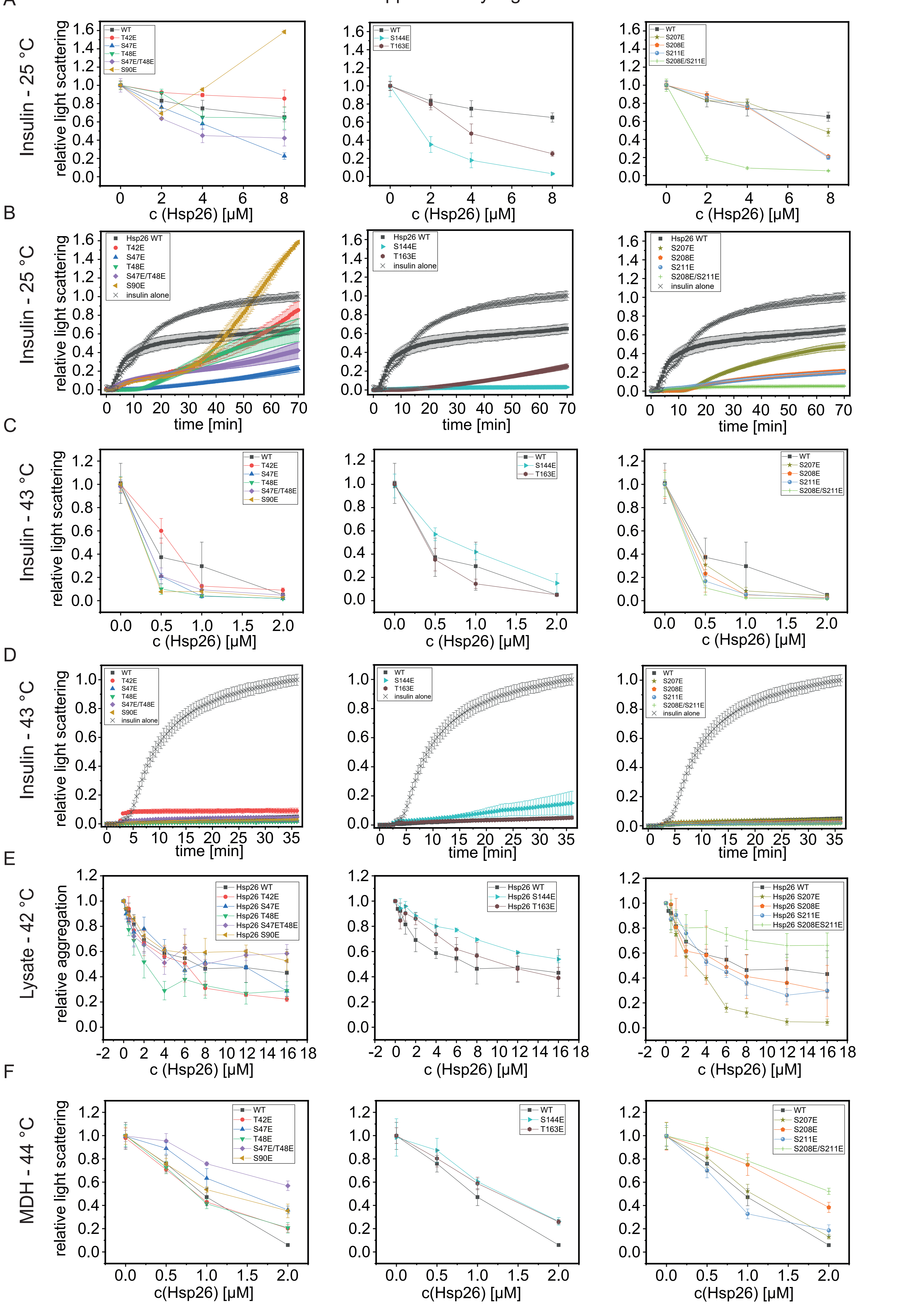

Supplementary Figure 1: Phospho-mimetic mutants tend to be more active at room temperature (belongs to figure 1).

A) Insulin (40 µM) assays were performed at 25 °C. Endpoints (70 min) of insulin assays performed with 0, 2, 4, 8 µM Hsp26 are plotted. Mean values and standard deviation are shown. N = 4 independent experiments for WT/0 µM, S47E/0 µM/4 µM/8 µM, T48E/0 µM//8 µM, S47E/T48E/0 µM, S90E/4 µM, T163E/4 µM/8 µM, S207E/0 µM/4 µM, S208E/0 µM, S211E/0 µM and S208E/S211E/0 µM/4 µM. N = 5 for S47E/T48E/4 µM/8 µM and N = 3 for all remaining samples. Aggregation of insulin was induced by the addition of 20 mM DTT. The assay was performed in PBS. Light scattering was followed in a photometer at 360 nm. The light scattering signal was normalized on the saturation value of the model substrate without chaperone.

B) Kinetics of the insulin assays performed at 25 °C as described in A with 8 µM Hsp26 variants. The color code can be found in the figure. Aggregation of insulin alone is indicated by black crosses.

C) Insulin (45 µM) assays were performed at 43 °C. Endpoints (36 min) of insulin assays performed with 0, 0.5, 1, 2 µM Hsp26 are plotted. Mean values and standard deviation are shown. N = 4 independent experiments for WT/0 µM/2 µM, T42E/2 µM, S47E/2 µM, T48E all concentrations, S47E/T48E/1 µM/2 µM, S90E/2 µM, S144E/0.5 µM/1 µM/2 µM, T163E/0 µM/2 µM, S207E/1 µM, S208E all replicates, S211E/0.5 µM/1 µM/2 µM and S208E/S211E/0 µM/0.5 µM/1 µM and N = 3 for all remaining samples. Aggregation of insulin was induced by the addition of 15 mM DTT. The assay was performed in 40 mM HEPES/KOH pH 7.5. Light scattering was followed in a photometer at 360 nm. The light scattering signal was normalized on the saturation value of the model substrate without chaperone.

D) Kinetics of the insulin assays performed at 43 °C as described in C with 2 µM Hsp26 variants. The color code can be found in the figure. Aggregation of insulin alone is indicated by black crosses.

E) The yeast lysate assay was performed with 0, 0.5, 1, 2, 4, 6, 8, 12, 16 µM Hsp26 at 42 °C and evaluated densitometrically with Image QuantTL (GE). All experiments were performed in at least triplicates. In the plots, mean values ± standard deviation are shown. N = 5 independent experiments and N = 4 for the S208E mutant and T42E/8 µM. N = 3 for all other samples

F) MDH (2 µM) assays were performed at 44 °C. Endpoints of MDH assays performed with 0, 0.5, 1, 2 µM Hsp26 are plotted. Mean values and standard deviation are shown. The assay was performed in PBS. Light scattering was followed in a photometer at 360 nm. The light scattering signal was normalized on the saturation value of the model substrate without chaperone. N = 3 independent experiments for S47E/0 µM, S47E/T48E/1 µM, S144E/0 µM /0.5 µM/1 µM, S207E/0 µM, S211E/0.5 µM/2 µM and S208E. N = 4 for all other samples.

Supplementary Figure 2

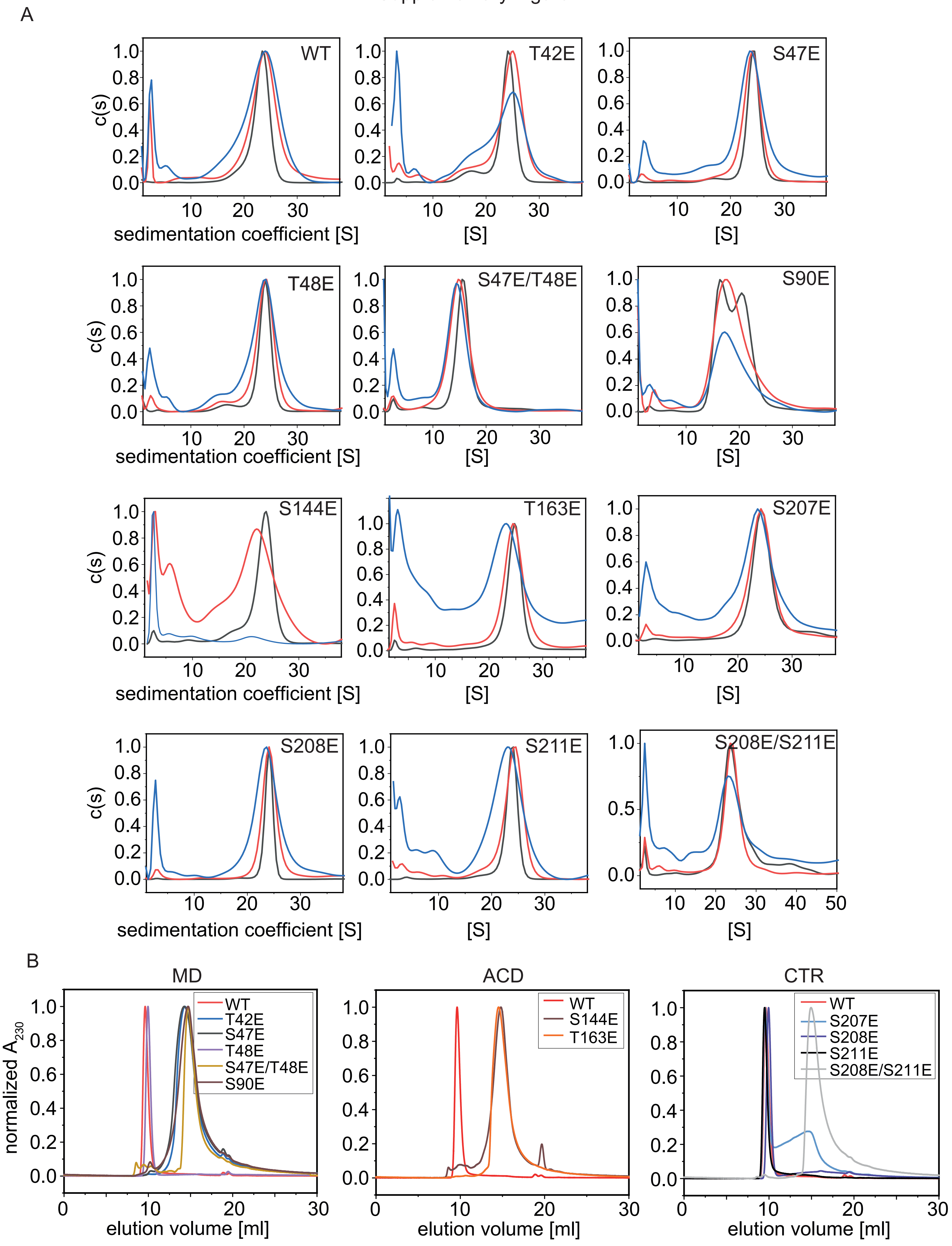

Supplementary Figure 2: Size of the oligomers strongly depends on the concentration (belongs to figure 2).

A) AUC runs of the different purified proteins were conducted at 23 µM (black), 7 µM (red) and 2.3 µM (blue) in PBS at 35,000 rpm (93,500 x g) and RT.

B) Determination of the oligomer size by SEC-HPLC. The protein samples (0.4 nmol) were separated with a Superdex200 increase 10/300GL (GE) column at a flow rate of 0.5 ml/min. Elution of the protein was followed at 230 nm. Normalized absorption values are plotted. The color code is shown in the figure.

Supplementary Figure 3

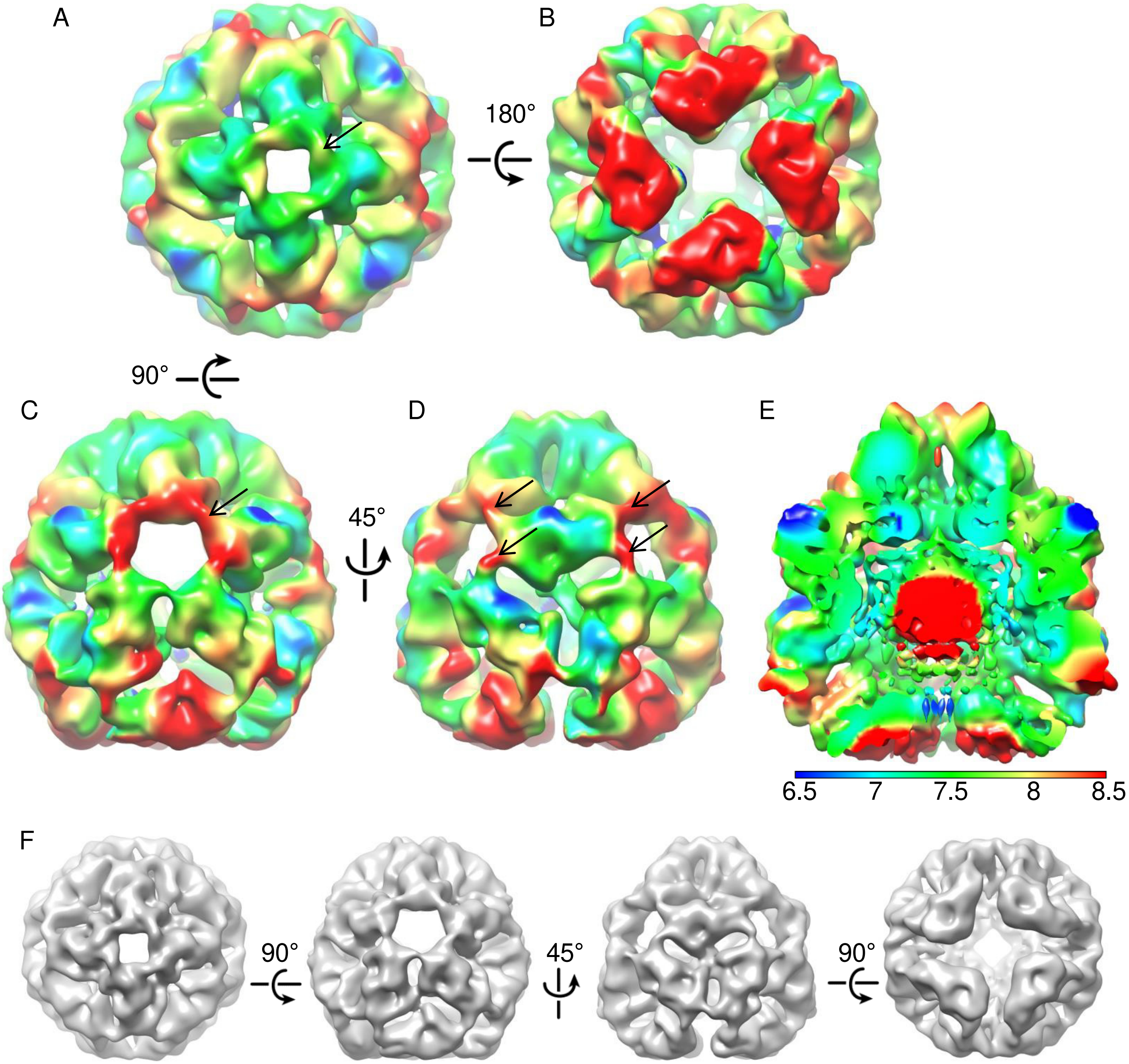

Supplementary Figure 3: Local resolution WT cryo-EM structure and reconstruction from yeast purified Hsp26 (belongs to figure 3).

A) to E) different views of the WT 40mer cryo-EM reconstruction colored by the local resolution, determined by cryosparc2. Arrows indicate some of the CTR interlinking different dimers. The scale bar (17 nm) shows the color code of the resolution in Å.

F) Surface views of the cryo-EM reconstruction of the Hsp26 WT protein purified from yeast. This is the same protein batch as used in White et al., 2006[1]. Views correspond to the views shown in Figure 3A.

Supplementary Figure 4

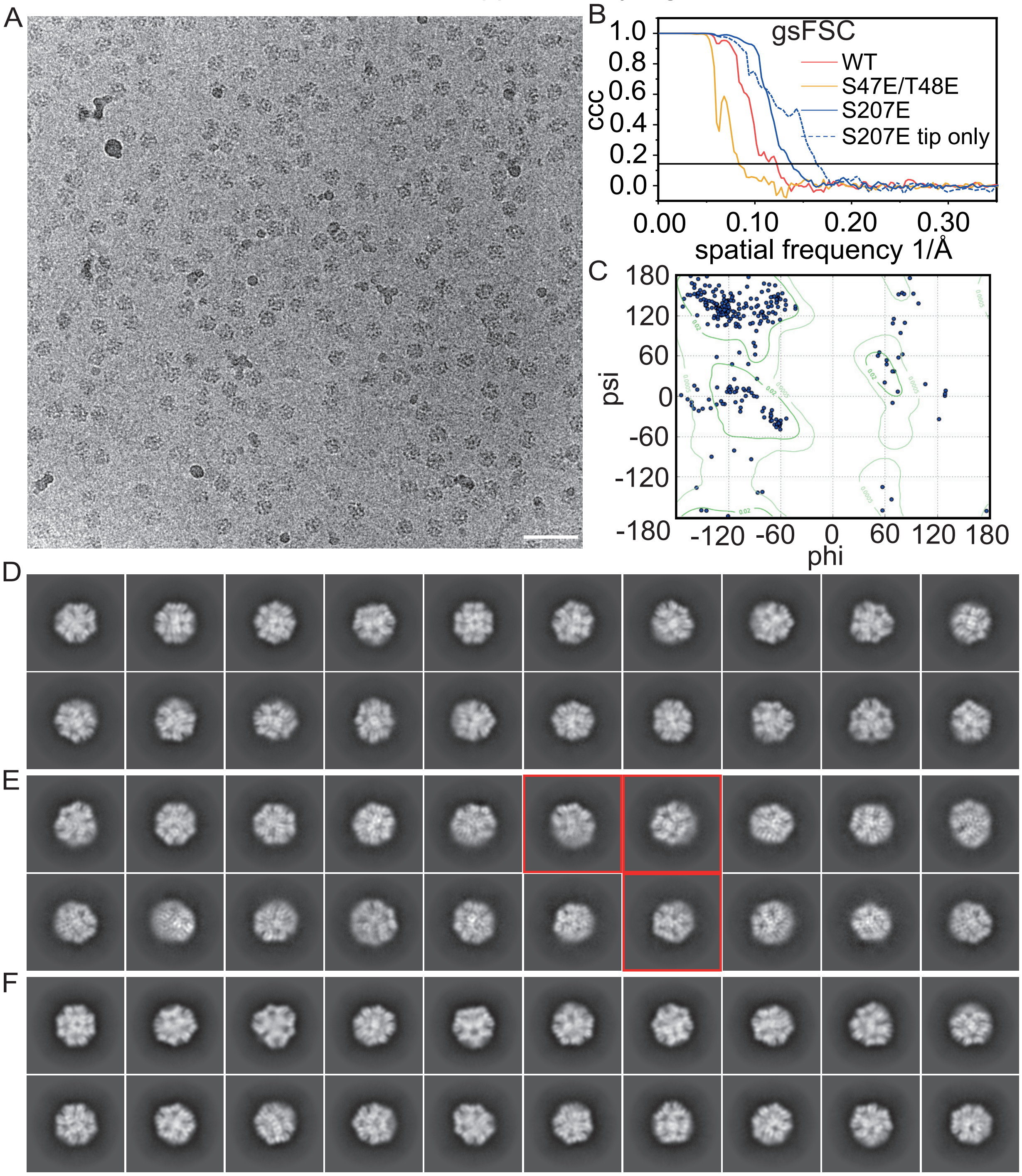

Supplementary Figure 4: EM data characterization (belongs to figure 3).

A) Typical cryo-EM micrograph of Hsp26, binned by factor 4 to increase protein visibility. The scale bar represents 50 nm.

B) Gold Standard Fourier Shell Correlation (FSC) computed using cryosparc (gold standard cut-off 0.1432 indicated with a black bar). This is the corrected FSC, thus calculated with a tight mask and phase randomization3 beyond the resolution determined by the FSC without masking. WT red: 8.2 Å; WT partial reconstruction of the top two rings red dashed 6.1 Å; S47E/T48E mutant beige: 12 Å; S207E mutant blue: 7.3 Å.

C) Ramachandran plot of the atomic model generated with Chimera.

D-F) The first 20 cryosparc 2D class averages sorted by number of particles per class. Box size 34 nm. D contains WT class averages. E contains class averages from the S47E/T48E mutant protein. The red frames highlight class averages which are only partly resolved (partly fuzzy). F contains class averages from the S207E mutant.

Supplementary Figure 5

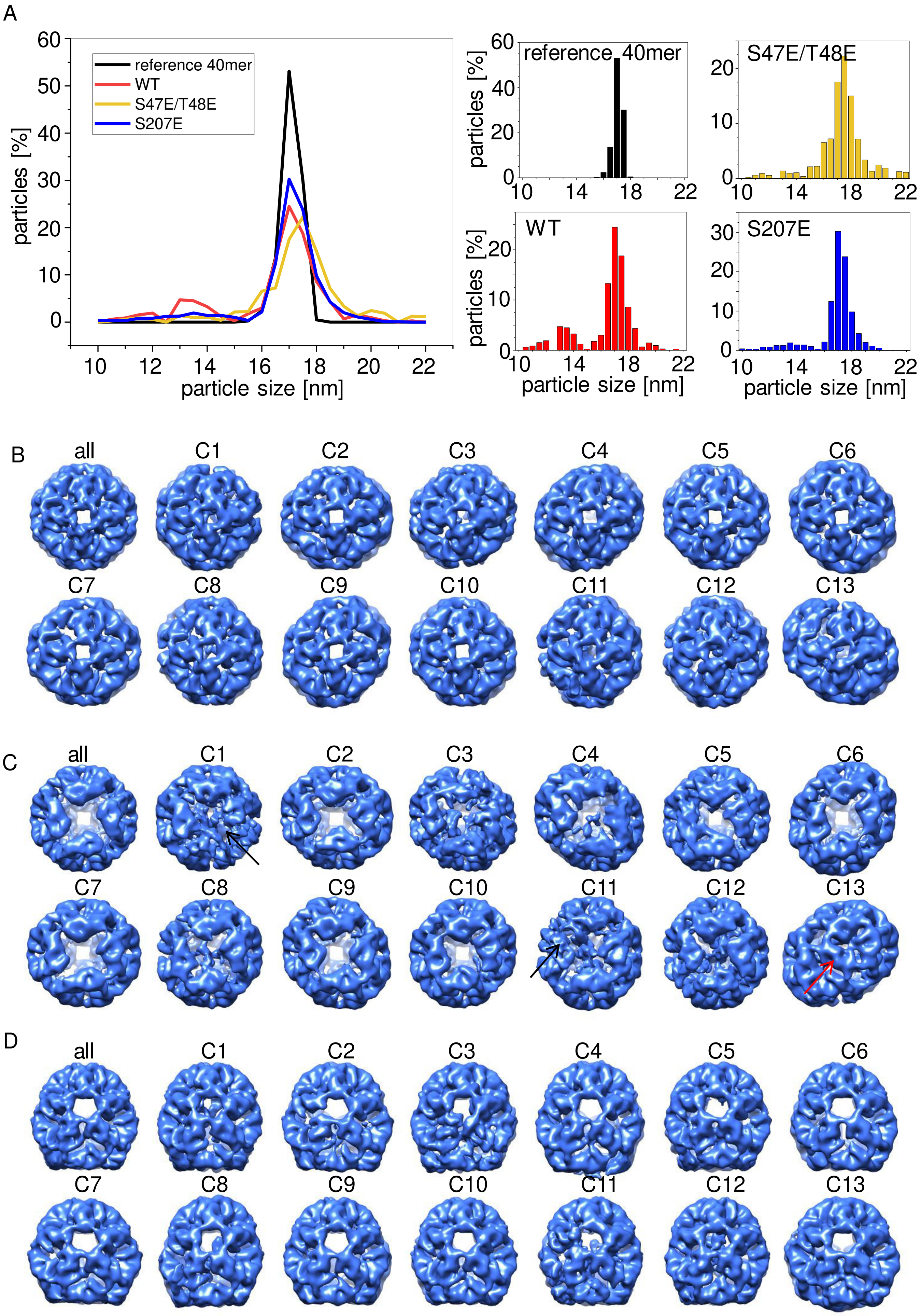

Supplementary Figure 5: 2D Analysis and 3D class averages illustrate the intrinsic heterogeneity of Hsp26.

A) Particle size distribution of the three datasets compared to the theoretical size distribution of a 40mer dataset (with perfectly random distribution of orientations; black). Measured ferret diameters were rounded to 0.5 nm and displayed as histogram in relation to the total number of picked particles. WT red, S47E/T48E beige, S207 blue and the theoretical 40mer black.

B) The S207E dataset was used for analysis, because it was more than 4 times as big as the WT particle dataset (Supplementary Table 2). These 3D class averages contain areas with density missing (black arrows) and with additional density (red arrow). The first class contains the symmetrized consensus structure containing all 393,415 particles from the best class of the ab initio reconstruction. The remaining class averages represent the 13 classes from the asymmetric heterogeneous refinement. B showing top views, C) showing views from the bottom (C13 is having an additional dimer bound indicated by the red arrow. Thus, it is a 42mer) and D) shows a side view of the consensus model and the 13 classes.

Figure S6

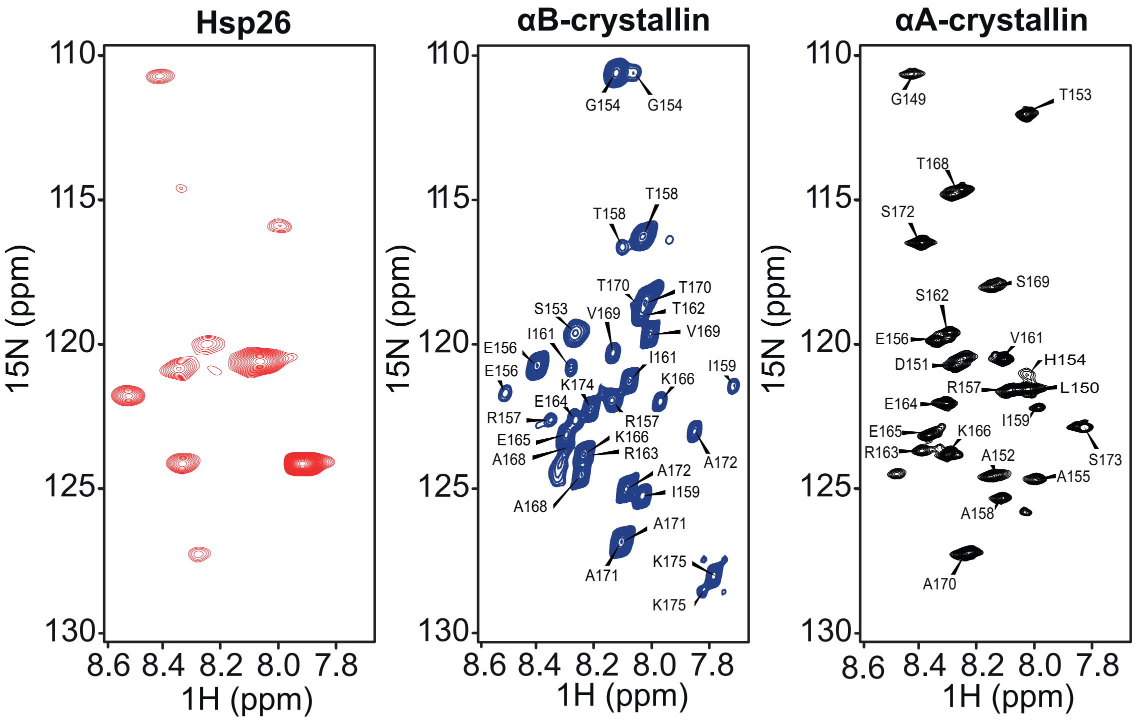

Supplementary Figure 6: Solution state NMR suggest a rigid CTR (belongs to figure 4).

Comparison of the solution-state 1H-15N HSQC spectra of Hsp26 at 37 °C (red), human αB-crystallin at 22 °C (blue) and human αA-crystallin at 22 °C (black). The observed resonances correspond to the CTR of each protein. The resonance assignment for αB-crystallin and αA-crystallin was performed in previous studies4,5. For Hsp26 only 10 peaks could be observed at 37 °C and the resolution did not suffice to obtain resonance assignments.

Supplementary Table 1: Melting temperatures of Hsp26 during thermal transitions in the CD and subunit exchange constants measured at 40 °C. Mean values und the standard deviation obtained from three replicates are shown.

**protein**	**T_M1 [°C]**	**T_M2 [°C]**	**$k_{exchange}$ x 10^{-3} [s^{-1}]**
Hsp26 WT	42.1 ± 0.1	69.5 ± 0.5	3.2 ± 0.4
Hsp26 T42E	39.0 ± 0.1	69.1 ± 0.8	3.4 ± 0.5
Hsp26 S47E	40.1 ± 0.2	68.6 ± 1.1	3.5 ± 0.3
Hsp26 T48E	39.3 ± 0.8	70.7 ± 0.8	3.4 ± 0.1
Hsp26 S47E/T48E	36.8 ± 0.6	71.8 ± 0.7	4.0 ± 0.3
Hsp26 S90E	37.8 ± 0.4	70.3 ± 0.6	4.0 ± 0.3
Hsp26 S144E	35.1 ± 0.1	69.0 ± 0.9	3.2 ± 0.1
Hsp26 T163E	34.6 ± 0.9	65.3 ± 1.1	3.6 ± 0.3
Hsp26 S207E	41.1 ± 0.1	68.7 ± 0.5	2.9 ± 0.2
Hsp26 S208E	39.6 ± 0.7	69.6 ± 0.5	2.7 ± 0.4
Hsp26 S211E	40.5 ± 0.7	69.8 ± 0.9	2.8 ± 0.3
Hsp26 S208E/S211E	37.7 ± 0.4	68.8 ± 0.4	3.6 ± 0.2

Supplementary Table 2: Cryo-EM data collection table.

	WT	WT yeast	S47E/T48E	S207E	S207E top rings
	EMD-12733	EMD-13748	EMD-12732	EMD-12731	EMD-12766
**pdb**					7OA6
**microscope**	Titan Krios	Titan Krios	Titan Krios	Titan Krios	Titan Krios
**voltage**	300	300	300	300	300
**magnification**	105k	105k	105k	105k	105k
**detector**	Gatan K2 summit	Gatan K2 summit	Gatan K2 summit	Gatan K2 summit	Gatan K2 summit
**electron dose (e^-/Å^2)**	28.3 and 30.8	32.1	15.4 and 22.2	43.8 and 46.1	43.8 and 46.1
**defocus range (µm)**	0.6 - 2.8	0.6 - 2.8	0.6 - 2.8	0.6 - 2.8	0.6 - 2.8
**pixel size (Å)**	1.33	1.33	1.33	1.33	1.33
**micrographs (no.)**	2,463	1,170	3,042	5,010	5,010
**symmetry imposed**	C4	C4	C4	C4	C4
**initial particle images (no.)**	160,621	102,640	279,160	688,419	688,419
**final particle images (no.)**	85,425	37,763	18,292	165,455	375,385
**relative abundance (%)**	53.2	36.8	6.6	24	54.52
**40mer particles (%)**	48.9	-	7.3	34.2	-
**box size (pixels)**	288	288	288	288	288
**map resolution (Å)**	8.2	9	12	7.3	6.1
**FSC threshold**	0.143	0.143	0.143	0.143	0.143

Supplementary Table 3: Primers used for cloning of Hsp26 mutants.

Hsp26 T42E fw	GTTAGCAAACGAGCCCGCAAAGGATTCTAC
Hsp26 T42E rev	TGACGTCTTGGTGCGTAG
Hsp26 S47E fw	CGCAAAGGATGAGACTGGCAAGG
Hsp26 S47E rev	GGTGTGTTTGCTAACTGAC
Hsp26 T48E fw	AAAGGATTCTGAGGGCAAGGAAGTTGCTAG
Hsp26 T48E rev	GCGGGTGTGTTTGCTAAC
Hsp26 S47E/T48E fw	CGCAAAGGATGAGGAGGGCAAGGAAG
Hsp26 S47E/T48E rev	GGTGTGTTTGCTAACTGAC
Hsp26 S90E fw	TTTCCCTAGAGAGGTCGCAGTTCCAG
Hsp26 S90E rev	CCGAAACCAGATGGGAAC
Hsp26 S144E fw	GAATGAAGAGGAGAAAGACAAGGTCAAG
Hsp26 S144E rev	AAGGTAGATGGAATTTCAC
Hsp26 T163E fw	GAGAGTCATCGAGTTGCCAGACTACC
Hsp26 T163E rev	TTGAACTTACCAGAGCTG
Hsp26 S207E fw	GATTGAGGTTGAGTCTCAAGAATCGTGG
Hsp26 S207E rev	TTCTTGACGTGGTTCTTAC
Hsp26 S208E fw	TGAGGTTTCTGAGCAAGAATCGTG
Hsp26 S208E rev	ATCTTCTTGACGTGGTTC
Hsp26 S211E fw	TTCTCAAGAAGAGTGGGGTAACTAAAAG
Hsp26 S211E rev	GAAACCTCAATCTTCTTGAC
Hsp26 S208E/S211E fw	GAAGAGTGGGGTAACTAAAAGCTTAATTAG
Hsp26 S208E/S211E rev	TTGCTCAGAAACCTCAATCTTCTTGAC
Hsp26 *Nco*1 fw	TACCATGGGTTCATTTAACAGTCCATTTTTTG
Hsp26 *Hind*III rev	TCCATAAGCTTTTAGTTACCCCACGAT
Hsp26 S6C fw	TTCATTTAACTGTCCATTTTTTGATTTC
Hsp26 S6C rev	CCCATGGTTAATTTCTCC

Supplementary References

1 White, H. E. *et al.* Multiple distinct assemblies reveal conformational flexibility in the small heat shock protein Hsp26. *Structure* **14**, 1197-1204, doi:https://doi.org/10.1016/j.str.2006.05.021 (2006).

2 Rosenthal, P. B. & Henderson, R. Optimal Determination of Particle Orientation, Absolute Hand, and Contrast Loss in Single-particle Electron Cryomicroscopy. *Journal of Molecular Biology* **333**, 721-745, doi:https://doi.org/10.1016/j.jmb.2003.07.013 (2003).

3 Chen, S. *et al.* High-resolution noise substitution to measure overfitting and validate resolution in 3D structure determination by single particle electron cryomicroscopy. *Ultramicroscopy* **135**, 24-35, doi:https://doi.org/10.1016/j.ultramic.2013.06.004 (2013).

4 Kaiser, C. J. O. *et al.* The structure and oxidation of the eye lens chaperone αA-crystallin. *Nature Structural & Molecular Biology* **26**, 1141-1150, doi:https://doi.org/10.1038/s41594-019-0332-9 (2019).

5 Mainz, A. *et al.* The chaperone αB-crystallin uses different interfaces to capture an amorphous and an amyloid client. *Nature Structural & Molecular Biology* **22**, 898-905, doi:10.1038/nsmb.3108 (2015).
