# Supplementary material for: Phosphorylation activates the yeast small heat shock protein Hsp26 by weakening domain contacts in the oligomer ensemble
Source: Nat Commun. 2021 Nov 18;12:6697. doi: 10.1038/s41467-021-27036-7 (PMC8602628; doi:10.1038/s41467-021-27036-7)
Supplement: Supplementary file 2 — Reporting summary [file 41467_2021_27036_MOESM2_ESM.pdf]

nature research

Corresponding author(s): Johannes Buchner, Sevil Weinkauf

Last updated by author(s): October 25, 2021

Reporting Summary

Nature Research wishes to improve the reproducibility of the work that we publish. This form provides structure for consistency and transparency in reporting. For further information on Nature Research policies, see our Editorial Policies and the Editorial Policy Checklist.

Statistics

For all statistical analyses, confirm that the following items are present in the figure legend, table legend, main text, or Methods section.

n/a	Confirmed	
☐	☒	The exact sample size (*n*) for each experimental group/condition, given as a discrete number and unit of measurement
☐	☒	A statement on whether measurements were taken from distinct samples or whether the same sample was measured repeatedly
☐	☒	The statistical test(s) used AND whether they are one- or two-sided<br>*Only common tests should be described solely by name; describe more complex techniques in the Methods section.*
☒	☐	A description of all covariates tested
☒	☐	A description of any assumptions or corrections, such as tests of normality and adjustment for multiple comparisons
☐	☒	A full description of the statistical parameters including central tendency (e.g. means) or other basic estimates (e.g. regression coefficient) AND variation (e.g. standard deviation) or associated estimates of uncertainty (e.g. confidence intervals)
☐	☒	For null hypothesis testing, the test statistic (e.g. *F*, *t*, *r*) with confidence intervals, effect sizes, degrees of freedom and *P* value noted<br>*Give P values as exact values whenever suitable.*
☒	☐	For Bayesian analysis, information on the choice of priors and Markov chain Monte Carlo settings
☒	☐	For hierarchical and complex designs, identification of the appropriate level for tests and full reporting of outcomes
☒	☐	Estimates of effect sizes (e.g. Cohen's *d*, Pearson's *r*), indicating how they were calculated

Our web collection on statistics for biologists contains articles on many of the points above.

Software and code

Policy information about availability of computer code

Data collection	MS data was collected with Xcalibur 3.0sp2. Photometric data were collected with CaryWinUV Bio pack 3.0. Fluorescence data were collected with FluorEssence (Origin7.5). CD data were collected with Pro-Data Chirascan. Cryo-EM: Automated image acquisition was performed with SerialEM. AUC data were collected with ProteomeLab6.0. The HPLC was operated with LCsolutions1.2. NMR: For data collection we used Bruker Topspin 3.5
Data analysis	Crosslink data were evaluated with Kojak 1.6.1 and Proxl. EM frames were aligned and dose weighted with MOTIONCOR2. Particles were picked with gautomatch 0.53 or with crYOLO 1.5.6 and the data processed in Relion 3.2.0 and cryosparc 3.2.0. 3D structures were visualized with Chimera 1.15. I-Tasser was employed to generate a homology model. MODELLER integration in Chimera was used to complete loops, e.g. connecting the CTR of different dimers. NAMD 2.12 was used for MDFF simulations and Phenix 1.19.2 for quality assessment. Origin2008b was used for plotting and statistical evaluations. Sedfit was used for AUC evaluation. Densiometric analysis was performed with ImageQuantTL. NMR: Bruker Topspin 4.0.6 and CcpNmr Analysis V2; Gimp 2.10.22 (TEM); Microsoft Office 2016; Adobe Illustrator CS5.

For manuscripts utilizing custom algorithms or software that are central to the research but not yet described in published literature, software must be made available to editors and reviewers. We strongly encourage code deposition in a community repository (e.g. GitHub). See the Nature Research guidelines for submitting code & software for further information.

Data

Policy information about availability of data

All manuscripts must include a data availability statement. This statement should provide the following information, where applicable:

- Accession codes, unique identifiers, or web links for publicly available datasets
- A list of figures that have associated raw data
- A description of any restrictions on data availability

Cryo-EM density maps have been deposited in the EMBD under the accession codes EMD-12773 (WT), EMD-13748 (WT yeast), EMD-12772 (S47E/T48E), EMD-12771 (S207E) and EMD-12766 (map of the top two rings used for flexible fitting).
The coordinates for the atomic model have been deposited in the PDB database under accession code 7OA6.
The mass spectrometry proteomics data have been deposited to the ProteomeXchange Consortium via the PRIDE partner repository with the dataset identifier PXD025314.
Source data are provided with this paper.

Field-specific reporting

Please select the one below that is the best fit for your research. If you are not sure, read the appropriate sections before making your selection.

☒ Life sciences ☐ Behavioural & social sciences ☐ Ecological, evolutionary & environmental sciences

For a reference copy of the document with all sections, see nature.com/documents/nr-reporting-summary-flat.pdf

Life sciences study design

All studies must disclose on these points even when the disclosure is negative.

Sample size	n was 3. This is the standard number of replicates in biochemical experiments
Data exclusions	No data were excluded.
Replication	the experiments were routinely performed in triplicates as usual in the life sciences
Randomization	Biochemical and structual biology experiments do not need to be randomized
Blinding	Blinding is not performed in structural biology and biochemical experiments

Reporting for specific materials, systems and methods

We require information from authors about some types of materials, experimental systems and methods used in many studies. Here, indicate whether each material, system or method listed is relevant to your study. If you are not sure if a list item applies to your research, read the appropriate section before selecting a response.

Materials & experimental systems

n/a	Involved in the study	
☒	☐	Antibodies
☒	☐	Eukaryotic cell lines
☒	☐	Palaeontology and archaeology
☒	☐	Animals and other organisms
☒	☐	Human research participants
☒	☐	Clinical data
☒	☐	Dual use research of concern

Methods

n/a	Involved in the study	
☒	☐	ChIP-seq
☒	☐	Flow cytometry
☒	☐	MRI-based neuroimaging
